# Structural Stability and Kinetics of Hydrogenation of β‑Tantalum at Low Temperatures

**DOI:** 10.1021/acs.jpcc.5c05265

**Published:** 2025-10-16

**Authors:** Ziqing Yuan, Herman Schreuders, Ewout Voorrips, Robert Dankelman, Roger M. Groves, Bernard Dam, Lars J. Bannenberg

**Affiliations:** † Faculty of Applied Sciences, 145265 Delft University of Technology, Mekelweg 15, Delft 2629 JB, The Netherlands; ‡ Faculty of Aerospace Engineering, 312572 Delft University of Technology, Kluyverweg 1, Delft 2629 HS, The Netherlands

## Abstract

The development of reliable hydrogen sensing materials for subzero environments is crucial for aviation, cryogenic storage, and hydrogen infrastructure applications. In this study, we investigate tetragonal β-tantalum (β-Ta) thin films at −60 °C to assess their potential for optical hydrogen sensing. *In situ* X-ray diffraction (XRD) measurements reveal a reversible lattice expansion upon hydrogen exposure, with β-Ta exhibiting a smaller volumetric expansion compared to α-Ta, indicating lower hydrogen solubility. Optical transmission measurements demonstrate a monotonic and fully reversible optical response across a range of hydrogen pressures, free of any hysteresis. However, β-Ta exhibits prolonged response times at low temperatures due to diffusion-limited kinetics, as confirmed by power-law response rate analysis and direct diffusion front measurements. Although β-Ta offers a temperature-independent resolution and structural robustness, its slower response time suggests the need for further microstructural optimizations to enhance hydrogen diffusion.

## Graphical abstract

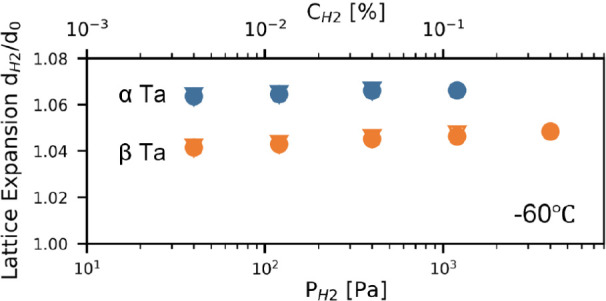

## 1 Introduction

The transition to a hydrogen-based energy economy is essential to reduce carbon emissions and meet global energy demands. Hydrogen, as a clean energy carrier, holds significant promise due to its high energy density and clean combustion, offering an ideal alternative to fossil fuels in achieving a net CO_2_-neutral society.^1–4^ However, its unique properties also introduce challenges related to safety and monitoring. Hydrogen’s small molecular size and high diffusivity make it prone to leakage, while its flammability at concentrations as low as 4% in air poses potential hazards.^5,6^ The ability to reliably and rapidly detect hydrogen is thus crucial for ensuring safe operation across various applications, including hydrogen-powered vehicles, fuel cells, and infrastructure systems.^7^ In addition, even the smallest leakages should be prevented as hydrogen is an indirect greenhouse gas prolonging the lifetime of e.g., CH_4_.^8^

In particular, hydrogen sensing technologies are critical for use in extreme environments such as arctic regions and aviation applications, where temperatures can fall to −60 °C^9,10^ and the total pressure ranges from 200 mbar ≤ *P*_tot_ ≤ 1.1 bar. For such conditions, sensors must reliably detect hydrogen in the concentration range of 0.1%–4%, corresponding to partial pressures of $20\leq P_{H_{2}}\leq 5000\;\mathrm{Pa}$ according to $P_{H_{2}}=C_{H_{2}}P_{\mathrm{tot}}$ . These conditions exacerbate the challenges of hydrogen detection and limit the functionality of conventional sensors of a catalytic, thermal conductivity, and electrochemical nature.^11^ Catalytic sensors require oxygen and elevated temperatures, making them unsuitable in oxygen-deprived or cold environments.^12^ Electrochemical sensors fail at low temperatures due to freezing electrolytes.^13^ Thermal conductivity sensors struggle with unstable thermal gradients, slower heat transfer, and interference from ice formation or reduced thermal conductivity differences in mixed gases.^13–15^ Across these technologies, reliance on electrical currents also introduces risks of electromagnetic interference and sparking—a critical hazard with hydrogen’s flammability at concentrations as low as 4% in air.^16^

Optical hydrogen sensors, including those based on metal–organic frameworks (MOFs), oxides, and metal hydrides, offer a robust solution for subzero hydrogen sensing.^17–21^ Optical metal hydride sensors are particularly promising, as they utilize the reversible absorption and release of hydrogen upon exposure to a hydrogen-containing atmosphere to induce measurable changes in optical properties like reflectivity or transmission.^21^ These sensors ensure high selectivity, a wide detection range, and precise performance even in extreme conditions.^22–28^ Additionally, their potential for miniaturization makes them ideal for integration into aviation systems and compact hydrogen storage setups.^1,29^

Among the various materials investigated for optical metal hydride hydrogen sensing, tantalum (Ta) and its alloys have demonstrated exceptional performance due to their ability to combine a wide sensing range, hysteresis-free operation, and swift response times, even under extreme subzero conditions.^22–25,28^ Recent studies confirm that tantalum-based materials, such as pure α-Ta which crystallizing in a body-centered cubic structure and its alloys with palladium (Ta_88_Pd_12_) and ruthenium (Ta_88_Ru_12_), maintain operational stability and provide sufficient optical contrast for hydrogen detection down to −60 °C.^28^ Notably, Ta_88_Ru_12_ exhibits the largest optical contrast and the fastest response time of just 6 s at −60 °C upon exposure to 3% hydrogen (3000 Pa), making it a leading material for hydrogen sensing in extreme environments.^28^ These properties, coupled with robust resistance to phase transitions and plastic deformation, establish tantalum-based sensors as reliable solutions for applications such as aviation and arctic hydrogen infrastructure.^22–25,28,30^

Despite impressive sensing capabilities at −60 °C, the response time, while adequate for some applications, remains a limiting factor that should ideally be improved. Hydrogen absorption and transport in tantalum-based thin films are governed by two primary mechanisms: (i) dissociation of hydrogen molecules on the surface and (ii) diffusion of hydrogen through the layer material.^28,30,31^ Research shows that even at −60 °C, the hydrogenation kinetics in α-Ta is restricted mainly by surface-limited processes, such as the dissociation step.^28^ Therefore, it would be beneficial to consider alternatives that absorb fewer hydrogen atoms to reach equilibrium at a given temperature and environmental hydrogen partial pressure/concentration.

One interesting candidate is metastable tetragonal β-tantalum (β-Ta), which exists only as a thin film. The tetragonal β-Ta structure offers excellent hydrogen sensing properties, including high optical contrast, reversible hydrogen absorption, and a broad dynamic range spanning several orders of magnitude in hydrogen pressure at room temperature.^25^ Most importantly, in this context, is its reduced hydrogen solubility compared to α-Ta, with a hydrogen-to-metal ratio of approximately *x*  ≈  0.5 at $P_{H_{2}}=24,600\;\mathrm{Pa}$ , compared to *x* ≈  0.7 for α-Ta. Consequently, the lattice expansion of β-Ta upon hydrogenation is lower, with a thickness increase of about 5%, whereas α-Ta expands by approximately 7% at the same hydrogen pressure.^25^ This reduced hydrogen absorption is particularly important as it reduces the lattice expansion, thereby reducing mechanical strain and improving structural stability. These properties raise an important question: can β-Ta still enable hysteresis-free hydrogen sensing at −60 °C? Will the reduced lattice expansion observed at room temperature persist under subzero conditions, and if so, could this help alleviate kinetic limitations arising from dissociation-limited surface reactions? If fewer hydrogen atoms are required to reach equilibrium, the kinetic bottleneck imposed by the dissociation step may be relaxed, potentially enabling faster hydrogen uptake even when dissociation kinetics are intrinsically slow. However, the distinct crystal structure of β-Ta may also affect hydrogen diffusion, introducing new kinetic constraints. It thus remains an open question whether β-Ta offers intrinsic advantages for hydrogen uptake under low temperature conditions.

Building upon these considerations, in this work, we investigate the fundamental hydrogenation behavior of thin film β-Ta at −60 °C, with particular attention to structural stability, optical reversibility, and kinetic limitations. Using a combination of *in situ* X-ray diffraction (XRD), optical transmission analysis, and direct in-plane diffusion measurements, we evaluate the intrinsic diffusion behavior in β-Ta and compare it directly to α-Ta. Our findings demonstrate that, while β-Ta maintains structural integrity and fully reversible lattice expansion at subzero temperatures, it exhibits significantly slower response times due to diffusion-limited hydrogen transport. These results establish β-Ta as a valuable platform for exploring diffusion phenomena in hydrogenated thin films under extreme temperature conditions.

## 2 Experimental Section

### 2.1 Sample Fabrication

The thin film samples shown in Figure S1 were fabricated on 10 × 10 mm^2^ quartz substrates (Mateck GmbH, Jülich, Germany) with a thickness of 0.5 mm and a surface roughness below 0.4 nm. The film structure consists of a 20 nm β-Ta metal hydride sensing layer, a 5 nm Pd_60_Au_40_ catalyst layer to enhance hydrogenation kinetics and mitigate oxidation, and a 30 nm polytetrafluoroethylene (PTFE) layer to improve hydrogen absorption kinetics while protecting the catalyst from contamination.

All layers were deposited using magnetron sputtering in an ultrahigh vacuum chamber (AJA Int.) with a base pressure of 10^–6^ Pa. The deposition process employed 5.08 cm (2 in.) targets with at least 99.9% purity (Mateck GmbH, Jülich, Germany) and was conducted under an argon pressure of 0.3 Pa. To ensure uniformity, the unheated substrates were continuously rotated during deposition. Thin film alloys were synthesized by co-sputtering two targets, adjusting the direct current (DC) power supply to achieve the desired composition. The deposition rates were 0.14 nm s^–1^ at 130 W DC for Ta, 0.11 nm s^–1^ at 50 W DC for Pd, and 0.10 nm s^–1^ at 30 W DC for Au. The PTFE layer was deposited via radiofrequency magnetron sputtering at 70 W, with a typical deposition rate of 0.029 nm s^–1^. A summary of deposition parameters is given in Table S1. The thickness and layered structure of the sample was confirmed by X-ray reflectometry (XRR) and X-ray diffraction (see below for experimental details) and the corresponding results are provided in Figure S2 and Figure S3. Surface characterization by tapping-mode atomic force microscopy (AFM) in Figure S4 indicates that the β-Ta-based multilayer exhibits a low peak-to-valley roughness (<2.5 nm), consistent with uniform film deposition. To further investigate the surface morphology and crystalline features of the films, scanning electron microscopy (SEM) was performed using a JSM-IT 700HR (JEOL, Germany) operated in high-vacuum mode with a secondary electron detector (SED). Images were acquired at accelerating voltages between 5–10 kV with magnifications up to 400,000 times. Owing to the presence of the 30 nm PTFE capping layer, which is amorphous, smooth, and relatively thick compared to the underlying metal layers, the β-Ta crystallites cannot be directly observed in the capped samples from Figure S5a). To address this, additional samples without the PTFE layer were fabricated under identical sputtering conditions. The corresponding SEM result in Figure S5b) clearly reveals the fine-grained structure of the β-Ta layer. These observations are consistent with the AFM and the crystalline nature of the metallic films, while also highlighting the protective role of the PTFE overlayer in the capped devices. In addition, the SEM images show the absence of any large cracks, micropores, or punctures in the film.

### 2.2 Structural Measurements

To investigate the structural behavior of the thin films at temperatures as low as −60 °C, we utilized a custom-designed *in situ* XRD setup capable of operating under controlled low-temperature and hydrogen atmosphere conditions, as detailed in ref 28. The system is based on a TTK 450 low-temperature chamber (Anton Paar, Graz, Austria), equipped with liquid-nitrogen cooling and a base pressure of 10^–3^ mbar. A schematic of the setup is provided in the Supporting Information (Figure S6).

The chamber was mounted on a Panalytical X’pert diffractometer (Almelo, The Netherlands) and used in Bragg–Brentano geometry over the range 30° ≤ 2θ ≤ 90°, employing a Cu–Kα X-ray source (λ = 0.1542 nm). Accurate sample height alignment was performed at room temperature by maximizing the peak intensity, after which the system automatically accounted for thermal expansion effects during temperature variations.

Hydrogen partial pressure control was achieved by adjusting the absolute pressure of a 4% H_2_ in He mixture (Linde Gas Benelux BV, Dieren, The Netherlands) using a pressure controller (MKS Type 250E, Andover, MA, USA) in conjunction with a solenoidal inlet valve and a Baratron pressure transducer (MKS120AD, Andover, MA, USA). The flow was regulated via a mass flow controller (Bronkhorst F-201CV-200-AAD-33-V EL Flow Select, Ruurlo, The Netherlands), with a parallel solenoidal outlet valve ensuring sufficient flow at low pressures. A custom LabVIEW program controlled and recorded gas flow and pressure in real time.

For room-temperature structural characterization, XRR and *ex situ* XRD measurements were carried out using a Bruker D8 Discover diffractometer (Bruker AXS GmbH, Karlsruhe, Germany) equipped with a LYNXEYE XE detector and a Cu Kα X-ray source (λ = 0.1542 nm). The resolution of the XRD, as determined by measuring a corundum reference sample, was approximately fwhm = 0.05° at 2θ = 25°, i.e., in the region of the (200) β-Ta and (110) α-Ta peaks. As such, the resolution has a negligible effect on the width of the diffraction peaks. For XRR measurements, an out-of-plane configuration with a Göbel mirror and a 0.1 mm exit slit on the primary side was used. On the secondary side, two 0.1 mm slits and the detector operating in 0D high count rate mode were employed. XRR data were analyzed using GenX3,^32,33^ and the extracted thickness, roughness, and density values for both as-prepared and hydrogenated samples are summarized in Table S2. The corresponding fits and scattering length density profiles are presented in Figure S2 and Figure S3.

### 2.3 Optical Measurements

To investigate hydrogen-induced changes in optical transmission between −60 °C and 20 °C, we employed a custom-built fiber-optic setup, described in detail in ref^28^. As shown in Figure S7, the system consists of a pressure cell placed in a temperature-controlled freezer (Elcold 11 Pro, Hørby, Denmark), equipped with two optical fiber feedthroughs: one delivers light from a halogen source (Ocean Optics HL-2000-FHSA, United States of America) to the sample, and the other collects the transmitted light and directs it to a spectrometer (Ocean Optics HR4000, United States of America), which provides a signal-to-noise ratio of 300:1 per acquisition and readout noise of 6 counts RMS. This allows us to confidently resolve the hydrogen-induced optical transmission changes even at low temperatures and low hydrogen concentrations.

The measured intensity $I_{H_{2}}$ was corrected for dark signal *I*_Dark_ and normalized to the transmission at 0.02% H_2_ (20 Pa), yielding the relative transmission:

1 $$\frac{T_{H_{2}}}{T_{0}}=\frac{I_{H_{2}}-I_{\mathrm{Dark}}}{I_{0.002\%}-I_{\mathrm{Dark}}}$$

Wavelengths between 650 and 660 nm were selected to ensure low noise and high source intensity. Hydrogen concentrations were controlled by mixing 5N Ar with either 4% or 100% H_2_ in Ar (Linde Gas Benelux BV, Dieren, The Netherlands), using three mass flow controllers (GF040CXXC, Brooks Instruments, Hatfield, PA, USA) regulated via LabVIEW. The hydrogen concentration $C_{H_{2}}$ in the resulting gas mixture was adjusted by setting the volumetric flow rates *Q*_Ar_ and $Q_{H_{2}}$ , corresponding to pure argon and the selected hydrogen-containing gas. It was calculated as

2 $$C_{H_{2}}=C_{\mathrm{gas}}\frac{Q_{H_{2}}}{Q_{H_{2}}+Q_{\mathrm{Ar}}}$$

where *C*_gas_ is the hydrogen concentration in the source gas (i.e., 4% or 100%). The total flow rate was fixed at 400 mL/min, and a check valve at the outlet maintained ambient pressure. Gas lines were evacuated between measurements using a vacuum pump (Adixen Drytel 1025, Pfeiffer Vacuum). A K-type thermocouple monitored the temperature inside the cell, remaining within ±2 °C. Measurements were taken only when the freezer compressor was inactive to avoid optical instability.

The response time was extracted from the normalized transmission curves following stepwise increases in hydrogen partial pressure $\left(24\leq P_{H_{2}}\leq 4000\mathrm{Pa}\right)$ , controlled by a pressure controller (MKS Type 250E, MKS Instruments, Andover, MA, USA) and monitored with a Baratron transducer (MKS). It is defined as the time required to reach 90% of the new equilibrium transmission level. Prior to each pressure step, the sample was held at 24 Pa for at least 5 min to ensure full hydrogen desorption and baseline stabilization.

## 3 Results

### 3.1 Structural Behavior of β-Ta under Hydrogen Exposure at −60 °C

Understanding the structural evolution of β-Ta when exposed to hydrogen is essential for assessing its applicability in hydrogen sensing. Previous studies at room temperature have shown that β-Ta undergoes a gradual, reversible hydrogen absorption without any hint of a first-order phase transition or plastic deformation.^25^ This is important as it enables a stable optical hydrogen sensing material that allows for hysteresis-free hydrogen sensing. However, Van’t Hoff’s law suggests that at a given partial hydrogen pressure/hydrogen concentration the hydrogen-to-metal ratio will increase when we lower the temperature. In addition, hydrogen-absorption induced phase transitions as well as plastic deformation are typically more likely to be observed at low temperatures.

Here, we investigate the structural effects of low-temperature hydrogen exposure by employing *in situ* XRD. Figure S8 presents the diffraction pattern for 30° ≤ 2θ ≤ 90° under conditions without hydrogen exposure, confirming that the β-Ta films are highly textured with (002) in the out of plane direction as in a previous study.^25^ Therefore, the *in situ* diffraction pattern of Figure 1, which shows measurements at −60 °C under various partial hydrogen pressures, only display the (002) reflection. We observe a peak shift toward lower diffraction angles with increasing partial hydrogen pressure, indicating a gradual lattice expansion due to hydrogen incorporation into the lattice.^34^ The absence of additional diffraction peaks across all investigated pressures suggests that β-Ta maintains a single-phase solid-solution behavior without undergoing a first-order phase transition. A slight decrease in peak amplitude and a concurrent increase in the width of the diffraction peak with increasing partial hydrogen pressure suggest the influence of microstrain and potential texture evolution within the β-Ta phase.^35^ Qualitatively similar results are obtained at −30 °C and 0 °C, where also no indication of any phase transition is found (Figure S9).

**Figure 1:**
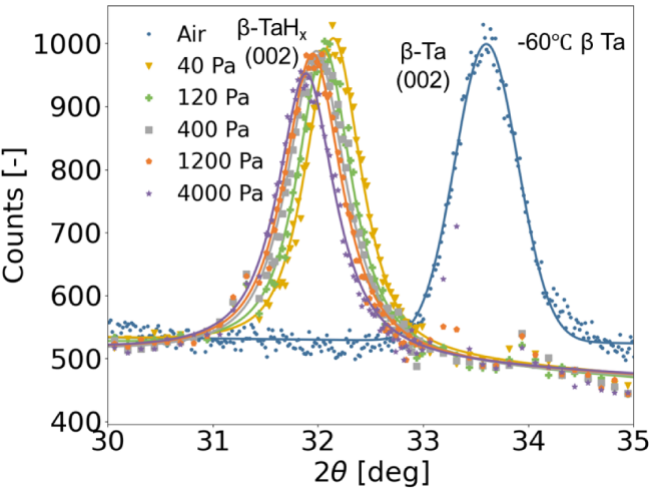
*In situ* X-ray diffraction patterns of a 20 nm β-Ta thin film with a 5 nm Pd_60_Au_40_ capping layer and a 30 nm PTFE protective layer on a quartz substrate, measured at −60 °C under the partial hydrogen pressures indicated in the legend. Diffraction peaks were fitted using a pseudo-Voigt function, which combines Gaussian and Lorentzian components to account for both instrumental and sample-induced broadening. The excellent fit quality (*R* ^2^ > 0.995) confirms the reliability of this approach.

Figure 2 presents the partial hydrogen pressure dependence of the out-of-plane lattice expansion that we obtain by fitting the diffraction patterns of Figure 1 and applying Bragg’s law. In this figure, we also display the lattice expansion of α-Ta^25,28^ which is highly textured with (110) in the out-of-plane direction, as well as previously obtained room-temperature data for β-Ta.^25^ In all cases, we normalize the *d*-spacing $d_{H_{2}}$ to the *d*-spacing of the lattice in the absence of hydrogen *d*_0_.

**Figure 2:**
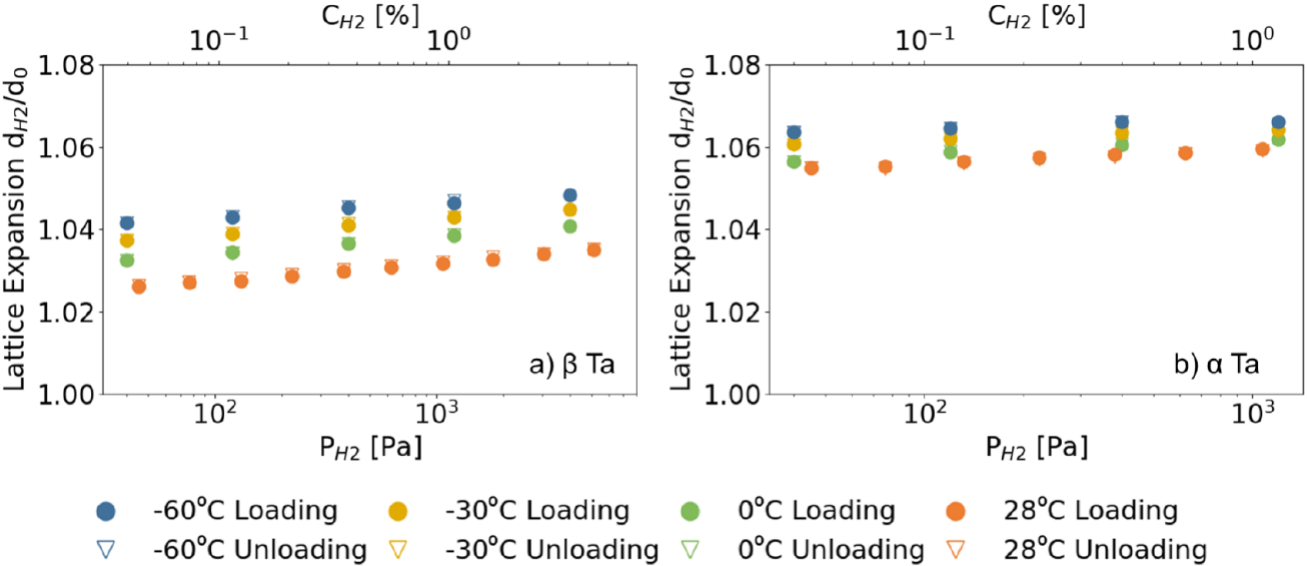
Partial hydrogen pressure dependence of a) the out-of-plane *d*_002_-spacing expansion in β-Ta and b) the *d*_110_-spacing expansion in α-Ta measured at the temperatures indicated in the legend. The *d*-spacing measured at a certain partial hydrogen pressure under thermal equilibrium is normalized to the *d*-spacing when no hydrogen is in the environment, *d*_0_. *d*_*hkl*_ is obtained by fitting the XRD data of Figure S9. The fitted peak position is converted to *d*_*hkl*_ using Bragg’s law. The open (closed) symbols indicate measurements performed after increasing (decreasing) the partial hydrogen pressure. The data for α-Ta at −60 °C, −30 °C and 0 °C is sourced from ref 28 The 28 °C data for β-Ta and α-Ta is sourced from ref 25.

Figure 2 shows that β-Ta exhibits a fully reversible hydrogenation that is free of any hysteresis in the partial hydrogen pressure range of $40\leq P_{H_{2}}\leq 4000\;\mathrm{Pa}$ , the measured *d*-spacing is the same after increasing or decreasing the hydrogen pressure/concentration, even at temperatures as low as −60 °C. This is consistent with the earlier notion that the lattice expands completely elastically and not plastically.

Remarkably, β-Ta exhibits significantly less lattice expansion than α-Ta across all investigated temperatures and hydrogen pressures. For example, at a temperature of −60 °C and $P_{H_{2}}=1200\;\mathrm{Pa}\;\left(C_{H_{2}}=1.2\%\right)$ , we observe a lattice expansion of 4.6% for β-Ta, substantially less than the 6.6% for α-Ta.^28^ The smaller lattice expansion suggests that, at a given partial hydrogen pressure or concentration, the hydrogen-to-metal ratio in the material is lower in β-Ta. This is advantageous, as it implies that less hydrogen needs to be absorbed, given that the increase in volume per absorbed hydrogen atom is similar for both materials.^25^ Furthermore, the moderate lattice expansion, combined with the absence of hysteresis, which suggests an elastic response, contributes to the material’s structural stability upon hydrogen exposure.^25,30^

### 3.2 Reversible Optical Response of β-Ta Thin Films to Hydrogen Exposure

#### 3.2.1 Monotoneous and Hysteresis-Free Transmission Response across Temperatures

For β-Ta to function effectively as an optical hydrogen sensing material, it must exhibit a stable, reversible and monotoneous relationship between the partial hydrogen pressure (concentration) and the optical transmission. A key requirement is that the transmission/reflection signal at a given hydrogen pressure and temperature remains identical after an increase or decrease in partial hydrogen pressure (concentration), ensuring a hysteresis-free sensing behavior. Additionally, rapid response times and high optical contrast are essential for practical hydrogen sensing applications. Previous studies have demonstrated that β-Ta gradually and reversibly absorbs hydrogen with increasing/decreasing hydrogen concentrations and induces a change in optical transmission and reflection at room temperature.^25^ The question, therefore, is whether the excellent hydrogen-sensing properties observed at room temperature extend to temperatures as low as −60 °C.

To assess the optical hydrogen sensing performance of β-Ta under extreme conditions, we conducted controlled hydrogenation experiments in which the hydrogen pressure was systematically increased and decreased in discrete steps while monitoring the corresponding optical transmission. Figure 3 shows an example of the normalized optical transmission response of a 20 nm β-Ta thin film (capped with 5 nm Pd_60_Au_40_ and a 30 nm PTFE protective layer) during these stepwise pressure variations at −60 °C. The lower panel shows the applied hydrogen pressure profile, which ranges from $P_{H_{2}}=20\;\mathrm{Pa}$ (0.002%) to 4000 Pa (4%), while the upper panel shows the corresponding optical transmission response in the 650–660 nm wavelength range, normalized to its initial optical transmission at 20 Pa $\left(T_{20\mathrm{Pa}}\right)$ . The optical transmission exhibits well-defined, reversible steps that closely follow the hydrogen pressure changes, with no observable hysteresis. This is evidenced by the near-perfect overlap of the optical response during increasing and decreasing pressure steps, as indicated by the dashed lines in Figure 3. Despite the relatively small optical modulation at −60 °C, the stepwise response is clearly distinguishable and exceeds the detection limit of the spectrometer. Furthermore, the response is monotonous, with each pressure increment leading to a proportional increase in transmission, and vice versa. These findings confirm that β-Ta provides a stable and predictable optical response to hydrogen exposure, making it a strong candidate for sensing applications in subzero environments.

**Figure 3:**
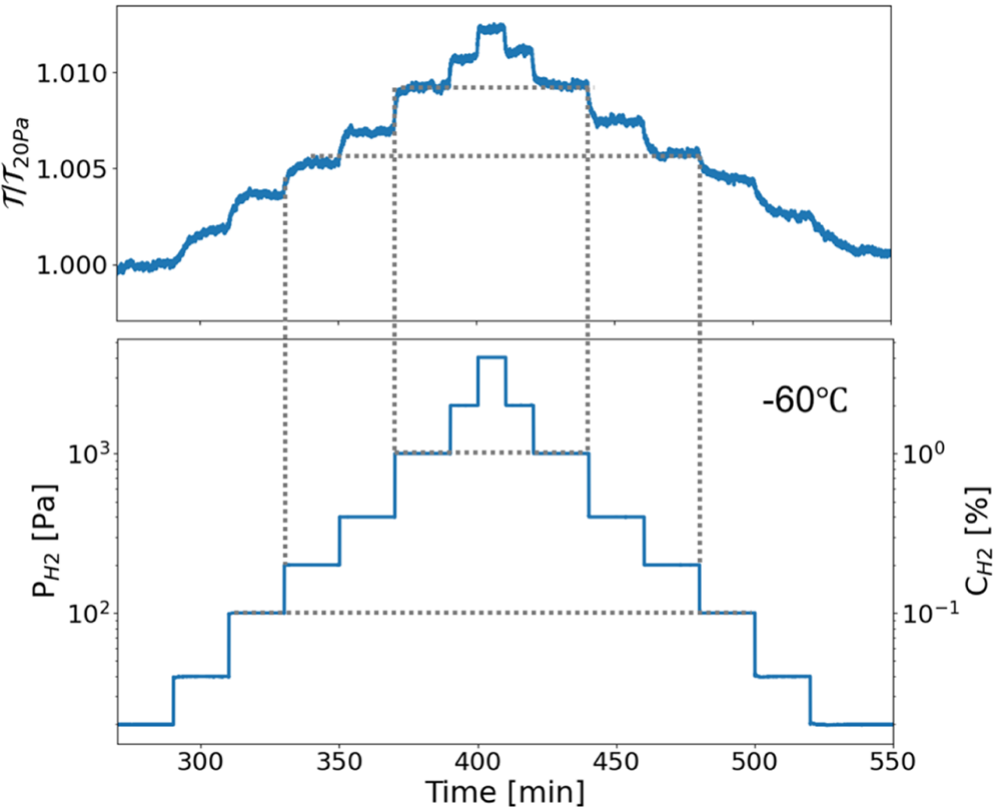
Time-dependent normalized optical transmission $\left(T/T_{\mathrm{20 Pa}}\right)$ response of a 20 nm β-Ta thin film (capped with 5 nm Pd_60_Au_40_ and a 30 nm PTFE protective layer) to various partial hydrogen pressures at −60 °C. The upper panel presents the corresponding optical transmission measured in the 650–660 nm wavelength range, normalized to the optical transmission at $P_{H_{2}}=20\;\mathrm{Pa}$ . The lower panel illustrates the stepwise variation of the partial hydrogen pressure $P_{H_{2}}$ over time. The dashed lines serve as guides to the eye, showing that the transmission follows an identical path during hydrogen absorption and desorption, indicative of a hysteresis-free response. The standard deviation of the optical signal in the 2% H_2_ region was determined to be σ = 8.45 × 10^–5^ at a sampling frequency of 0.5 Hz and wavelength range λ 650–660 nm. This corresponds to a sensitivity of $\Delta P_{H_{2}}/P_{H_{2}}=0.038$ . The detailed calculation is shown in Figure S19.

To further explore the normalized optical transmission $\left(T/T_{20\mathrm{Pa}}\right)$ of β-Ta under hydrogen exposure, we summarize the optical transmission measurements in Figure 4. Here, we plot the normalized optical transmission as a function of the partial hydrogen pressure/concentration. Each point in Figure 4 corresponds to the average transmission value at a given hydrogen pressure and temperature as measured following the protocol of Figure 3. The results confirm the monotonous relationship between the partial hydrogen pressure/concentration and the optical transmission. In addition, we observe that at this wavelength and layer thickness, α-Ta provides for all temperatures observed a larger optical response to a change in the partial hydrogen pressure, which implies a higher resolution than β-Ta. It is important to note, however, that the difference in sign of the slope of the normalized transmission.

**Figure 4:**
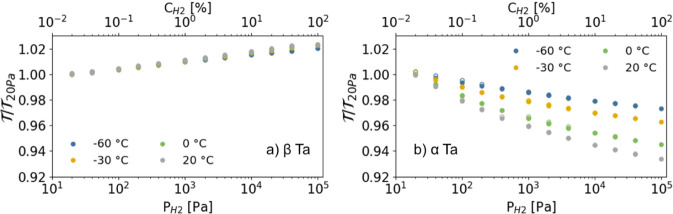
Normalized optical transmission $\left(T/T_{20\mathrm{Pa}}\right)$ of a) β-Ta and b) α-Ta as a function of partial hydrogen pressure $\left(P_{H_{2}}\right)$ at different temperatures (−60 °C, −30 °C, 0 °C and 20 °C). The transmission at each hydrogen pressure is normalized to the optical transmission at $P_{H_{2}}=20\;\mathrm{Pa}$ . Data for α-Ta is adapted from ref 28.

Remarkably, the slope of the normalized optical transmission $\left(T/T_{20\mathrm{Pa}}\right)$ of β-Ta remains nearly unchanged across all studied temperatures (−60 °C, −30 °C, 0 °C and 20 °C), indicating that its resolution is temperature-independent. This behavior aligns with the XRD data, which show that the normalized lattice expansion, referenced to the lowest measured $P_{H_{2}}$ , remains stable across different temperatures (Figure S10). In contrast, the slope for α-Ta varies significantly with temperature. The close similarity between the normalized optical transmission behavior of β-Ta and its structural response suggests that normalizing the transmission to a baseline pressure signal effectively enables temperature-independent resolution over a broad temperature range. This dramatically simplifies calibration for hydrogen sensing applications, enhancing both practical usability and reliability.

For hydrogen sensing materials, the relative change in optical transmission upon exposure to hydrogen is typically wavelength dependent. As such, selecting a proper wavelength can result in an increased resolution of the sensor. To this end, Figure S11 presents the transmission response of β-Ta across multiple spectral bands, highlighting its distinct wavelength-dependent behavior. Specifically, at longer wavelengths (e.g., 650–660 nm), hydrogenation results in an increase in transmission, whereas at shorter wavelengths (e.g., 520–530 nm), it leads to a decrease in transmission. This trend, which was previously observed at room temperature,^25^ is now confirmed at −60 °C. The results further indicate that the transmission change is most pronounced in the visible range, with the strongest optical contrast appearing at intermediate wavelengths (e.g., 650–730 nm), while a more moderate response is observed in the near-infrared region (e.g., 920–930 nm). Moreover, the optical contrast in the near-infrared region differs markedly between α- and β-Ta. For α-Ta, the 920–930 nm range exhibits the highest optical contrast within the measured wavelength range.

Notably, the opposite transmission changes, i.e., the decrease of the transmission to hydrogen exposure at shorter wavelengths and the increase at longer ones, can be used to suppress drift and increase sensitivity. Indeed, by measuring a short and long wavelength and subtracting the signal, one can reduce the drift while enhancing the amplitude of the optical response. In this way, the sensitivity, sensor stability are enhanced and background fluctuations are mitigated.^36^

#### 3.2.2 Cyclability of β-Ta Thin Films at −60 °C

To assess the long-term durability and reliability of β-Ta-based hydrogen sensors under cyclic operation, we conducted multiple hydrogen absorption/desorption cycles at both room temperature (20 °C) and −60 °C on a sample that was stored for 1 year in a desiccator which ensures a dry and stable environment. Figure 5a and c displays the time-resolved normalized optical transmission (τ/τ_0_) during repeated switching between 0.25% and 100% H_2_ concentrations. At both temperatures, the films show highly reproducible and reversible optical responses over consecutive cycles, demonstrating the hysteresis-free nature of the sensing behavior even at subzero conditions.

**Figure 5:**
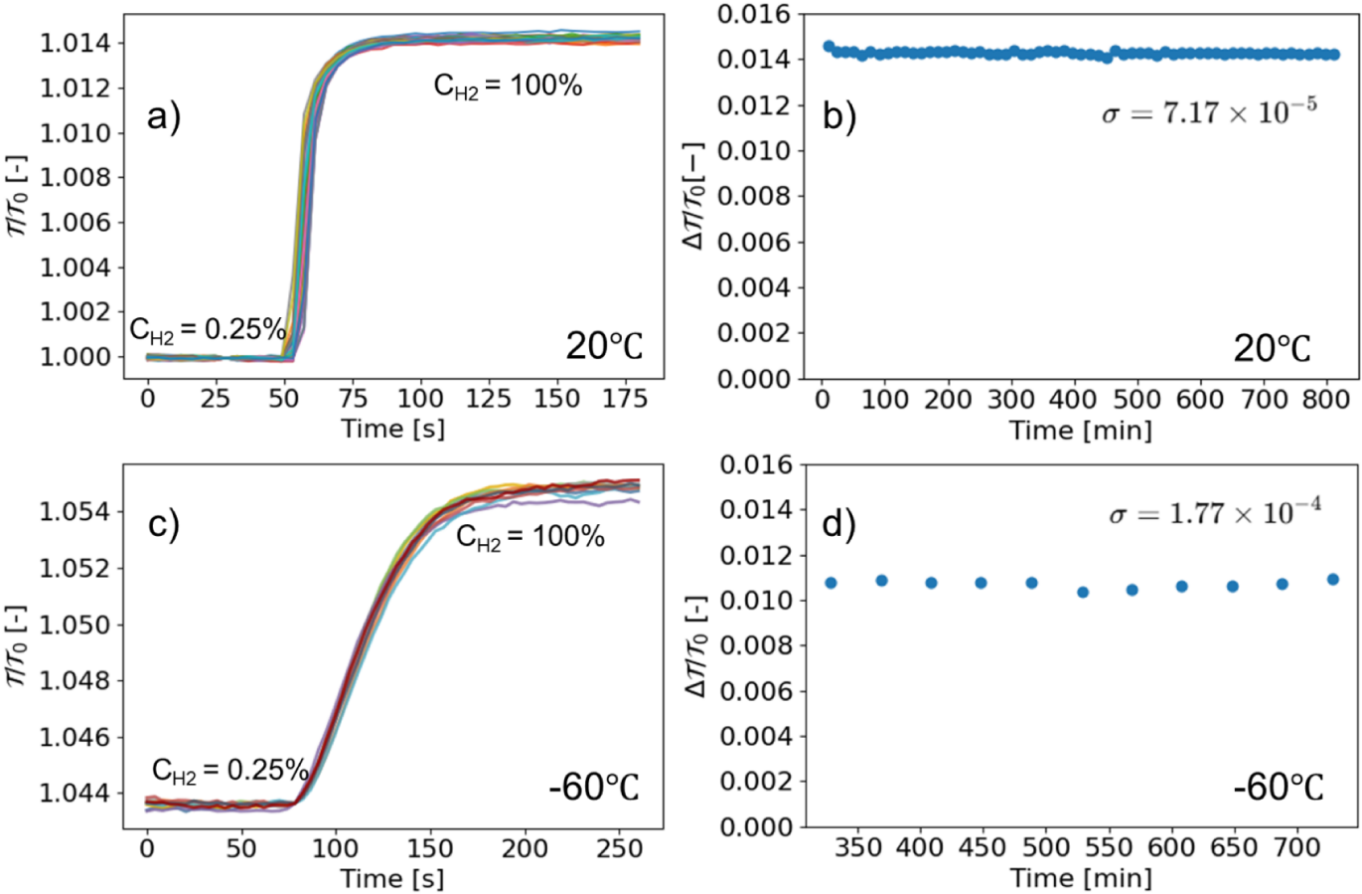
Optical transmission response of β-Ta thin films during repeated hydrogen absorption/desorption cycles at 20 °C and −60 °C. a) Normalized transmission τ/τ_0_ as a function of time for 61 cycles at 20 °C, the different colors in the figure indicate different cycles. b) Corresponding contrast values Δτ/τ_0_ versus time, with a standard deviation of σ = 7.17 × 10^–5^. c) Normalized transmission τ/τ_0_ over 10 cycles at −60 °C. d) Contrast values at −60 °C remain stable across cycles with σ = 1.77 × 10^–4^.

The amplitude stability of the hydrogen response was quantified by evaluating the optical contrast, defined as Δτ/τ_0_ = (τ_100%_ – τ_0.25%_)/τ_0_, across all cycles. As shown in Figure 5b and d, the contrast remains constant within experimental uncertainty throughout 61 cycles at 20 °C and 10 cycles at −60 °C. The corresponding standard deviations in Δτ/τ_0_ are 7.17 × 10^–5^ and 1.77 × 10^–4^, respectively. We employed XRD to further investigate the structural behavior related to long-term stability. The XRD pattern from Figure S12 confirm that the β-Ta remains in the same phase, exhibiting strong (002) texturing, even for the sample that had already been stored in a desiccator for 4 months after the cycling test. These results confirm that β-Ta thin films not only exhibit excellent optical reversibility but also retain their sensing performance over extended cycling.

### 3.3 Response Time and Response Rate of β-Ta

The response time of a hydrogen-sensing material is a crucial parameter, as it determines how quickly the sensor reacts to changes in the ambient hydrogen pressure. To measure the response time, we changed the partial hydrogen pressure from a reference pressure of $P_{H_{2}}=24\;\mathrm{Pa}$ (0.024%) to the partial hydrogen pressure of interest. We then define the response time *t*_90_ as the time it takes to reach 90% of its equilibrium state. The normalized response time curves can be found in Figure S13. Figure 6 compares the response time of β-Ta at various hydrogen pressures and temperatures with that of α-Ta from our previous study.^28^

**Figure 6:**
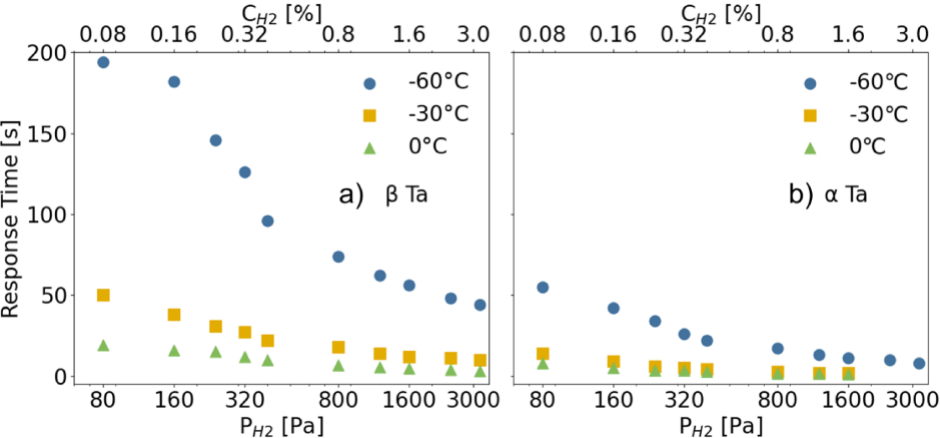
Response times of a) β-Ta and b) α-Ta as a function of partial hydrogen pressure $\left(P_{H_{2}}\right)$ at different temperatures (−60 °C, −30 °C, 0 °C).

For β-Ta (Figure 6a), the response time at −60 °C is significantly longer than at higher temperatures, indicating sluggish hydrogen uptake kinetics. At this temperature, it reaches 194 s at $P_{H_{2}}=80\;\mathrm{Pa}$ (0.08%) and 44 s at $P_{H_{2}}=3200\;\mathrm{Pa}$ (3.2%). As the temperature increases to −30 °C, the response time decreases substantially to 50 s at 80 Pa and 10 s at 3200 Pa. At 0 °C, hydrogen absorption is fastest, with response times of 19 s at 80 Pa and just 3 s at 3200 Pa, demonstrating significantly improved hydrogenation kinetics at elevated temperatures. In contrast, α-Ta (Figure 6b) exhibits consistently shorter response times than β-Ta across all temperatures and pressures. Even at −60 °C, its response remains well below that of β-Ta, reaching 55 s at 80 Pa and 8 s at 3200 Pa under identical conditions.

To understand why the kinetics of β-Ta are slower than those of α-Ta, we examine the partial hydrogen pressure dependence of the response rate. The response rate provides key insights into whether hydrogenation is primarily limited by surface effects or bulk diffusion. To this end, we fit the partial hydrogen pressure $P_{H_{2}}$ dependence of the response rate *R* to a power law according to

3 $$R=aP_{H_{2}}^{\gamma }$$

where the exponent γ can be used to characterize the dominant transport mechanism. Borgschulte et al.^31^ established that γ  ≈  1 indicates a surface-limited process dominated by hydrogen dissociation or other surface effects, while γ  ≈  0.5 suggests bulk diffusion as the rate-limiting step.

To quantify the response rate in this study, we use the relationship between the hydrogen-to-metal ratio and lattice expansion, denoted as $d_{H_{2}}/d_{0}$ , which directly reflects hydrogen incorporation into the lattice. While lattice expansion typically exhibits a nonlinear relationship with the hydrogen-to-metal ratio in most materials due to three-dimensional expansion, it has been shown to be linear in thin-film β-Ta.^25^ Therefore, the response rate can be expressed as

4 $$R\propto \frac{d_{H_{2}}/d_{0}}{t_{90}}$$

Figure 7a presents the response rate of β-Ta across different temperatures (−60 °C, −30 °C and 0 °C) and the fits of eq 3 to the data. The extracted values of γ are 0.53 ± 0.03 at 0 °C, 0.46 ± 0.01 at −30 °C, and 0.45 ± 0.02 at −60 °C, all close to 0.5. This behavior suggests that hydrogen uptake in β-Ta is predominantly diffusion-limited, in contrast to α-Ta (Figure 7 b), where the kinetics were found to be surface/dissociation limited, evidenced by the exponents close to 1.^28^ As in both samples a Pd_60_Au_40_ catalyst and PTFE protective layer was used, this would imply that diffusion in the α-Ta structure is much faster than in the β-Ta lattice.^28,37–39^

**Figure 7:**
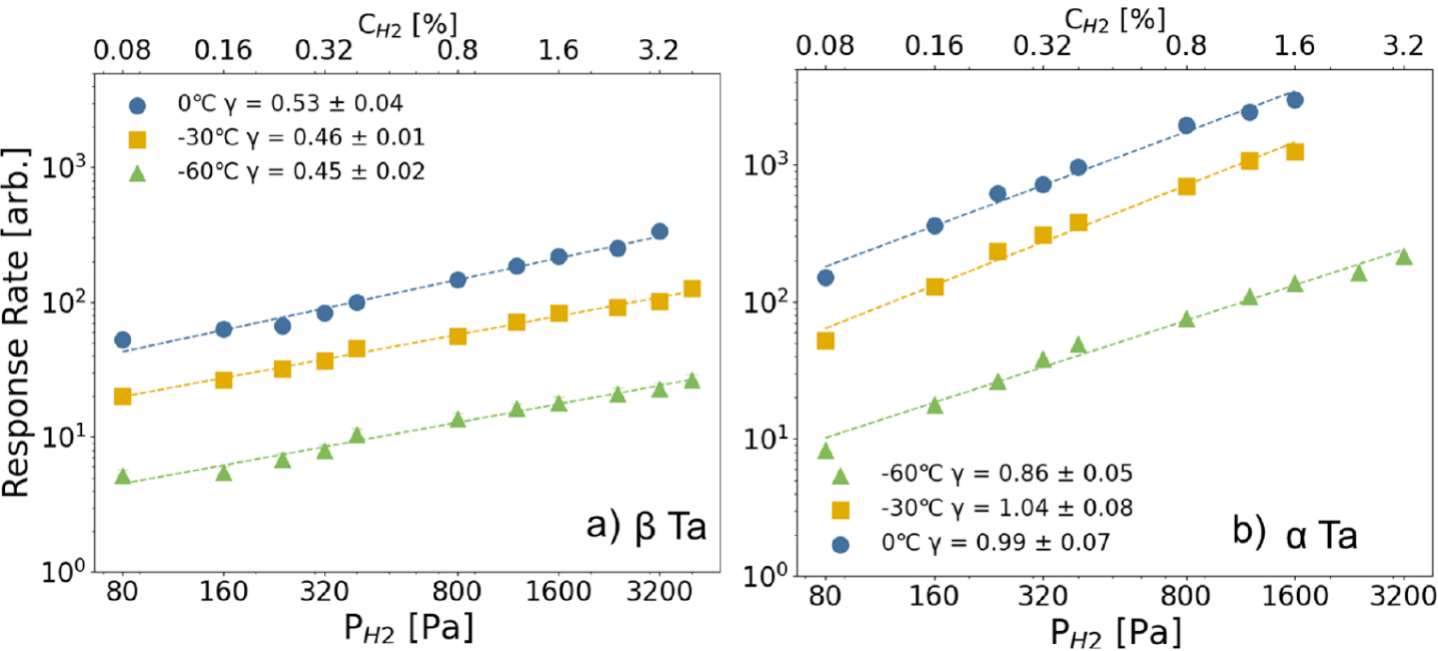
(a) Response rate calculated using eq 4 of β-Ta as a function of partial hydrogen pressure $\left(P_{H_{2}}\right)$ at different temperatures (−60 °C, −30 °C and 0 °C). b) Response rate of α-Ta as a function of partial hydrogen pressure $\left(P_{H_{2}}\right)$ at different temperatures (−60 °C, −30 °C and 0 °C), sourced from ref 28 The dashed lines indicate fits of the response rate at different temperatures to eq 3.

### 3.4 Hydrogen Diffusion Measurement in α-/β-Tantalum Thin Films at 28 °C

To demonstrate that hydrogen diffusion is the rate-limiting step in β-Ta and to enable a direct comparison with α-Ta, we conducted optical transmission measurements capable of tracking in-plane hydrogen diffusion, as demonstrated in ref 40 which served as the basis for our experimental design. For this purpose, a dual-phase diffusion sample was fabricated on a large-area 3 in. fused quartz wafer (Figure 8). A 4 nm Ti seed layer was deposited on one-half of the substrate using a shadow mask to promote the formation of α-Ta.^25^ Following mask removal, a uniform 80 nm Ta layer was sputtered across the entire wafer, resulting in the coexistence of α-Ta (on the Ti-coated side) and β-Ta (on the bare quartz side). The Ta layer was subsequently capped with a 60 nm Y layer, which serves both as a hydrogen diffusion indicator and an oxidation barrier. A hexagonally arranged array of 0.9 mm diameter and 20 nm thick Pd dots was then deposited using a stencil mask to act as catalytic hydrogen to serve as catalytic sites for hydrogen dissociation and entry. Detailed sputtering parameters for all layers are summarized in Table S3. The successful formation of distinct α-Ta and β-Ta regions was confirmed by XRD, as shown in Figure S14.

**Figure 8:**
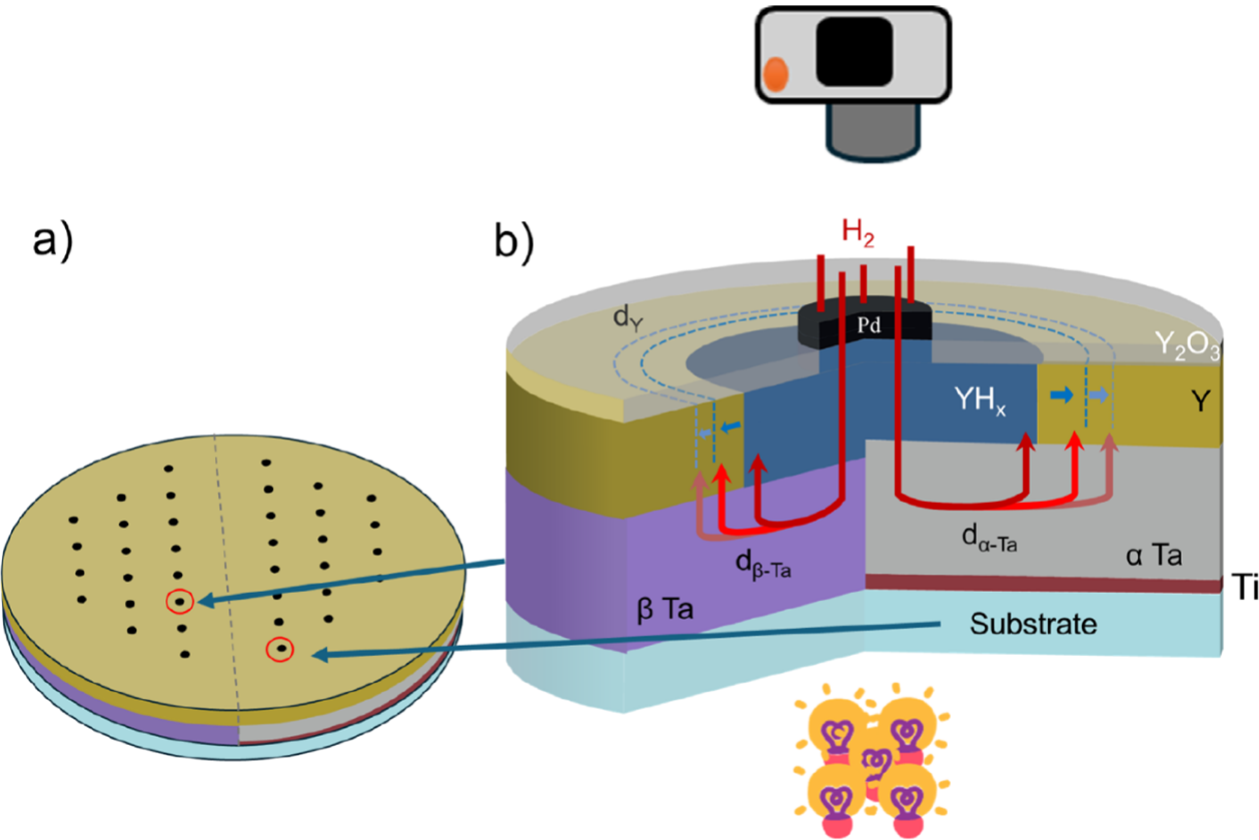
Schematic overview of the α-/β-Tantalum diffusion sample and the hydrogen diffusion principle. a) Illustration of the full sample fabricated on a 3-in. fused quartz wafer. A 4 nm Ti seed layer was deposited on the right half (red area) to promote the formation of α-Ta, while the left half remained without Ti, yielding β-Ta after subsequent deposition of an 80 nm Ta film across the entire wafer. The Ta layer was capped with 60 nm of Y, serving as both hydrogen diffusion indicator and oxidation barrier. A hexagonally arranged array of 0.9 mm diameter, 20 nm thick Pd dots was deposited to locally catalyze hydrogen dissociation. Note that the Pd dot arrangement shown here is a schematic representation and does not precisely reflect the actual hexagonal layout. b) Cross-sectional schematic of the diffusion mechanism. Hydrogen gas dissociates on the Pd dots, diffuses vertically into the Ta film, and then spreads laterally within the Ta layer, where its transport is orders of magnitude faster than in the YH_*x*_ layer. The hydrogen finally reacts with the Y capping layer to form optically visible yttrium hydride. By monitoring the time-resolved expansion of the yttrium hydride regions, the hydrogen diffusion in both α-Ta and β-Ta can be tracked optically. Adapted with permission from ref 22 Copyright 2019 Elsevier.

This technique to measure the in-plane diffusion utilizes the optical effect that when hydrogen diffuses through the tantalum layer the yttrium layer changes color. Hydrogen can only enter this sample through the Pd dots. As the diffusion in Yttrium $\left(D_{{\mathrm{YH}}_{x}}∼1\times 10^{-11}\;{\mathrm{cm}}^{2}\;s^{-1}\right)$ ^41^ is assumed to be significantly slower than through Ta (D_Ta_ ∼ 1 × 10^–6^ cm^2^ s^–1^),^22^ it occurs through the Ta layer. As Y absorbs hydrogen at an extremely low partial hydrogen pressure of $P_{H_{2}}\approx 1\times 10^{-22}\;\mathrm{Pa}$ ,^22^ it will directly absorb hydrogen when hydrogen can be supplied through the Ta layer. When Y absorbs the hydrogen and forms a hydride, it changes from a metal to an insulator. To visualize this, the sample was illuminated from below, and images were recorded at fixed intervals using a CCD camera positioned above (Figure 8). By tracking the radial expansion of the optically brightened regions surrounding each Pd dot, the lateral hydrogen diffusion kinetics in both α-Ta and β-Ta regions can be directly compared. The experiment was performed at 28 °C and at $P_{H_{2}}=1\;\mathrm{bar}\;\left(C_{H_{2}}=100\%\right)$ . Full details of the setup are available in Figure S15.

Figure 9 presents the time evolution of the hydrogen diffusion fronts in both α-Ta and β-Ta regions. The faster radial diffusion front propagation in α-Ta provides direct evidence for its higher hydrogen mobility relative to β-Ta. A more detailed illustration of the region selection and diffusion front diameter extraction based on optical images is provided in theSupporting Information (Figure S16). After 20 h of hydrogen exposure, the diffusion front in α-Ta moved from 0.90 mm to 3.27 mm in diameter, while in β-Ta it expanded from 0.90 mm to 1.25 mm. The diffusion front in α-Ta thus moved nearly seven times further outward than in β-Ta, clearly illustrating the significantly higher hydrogen mobility in the α-phase.

**Figure 9:**
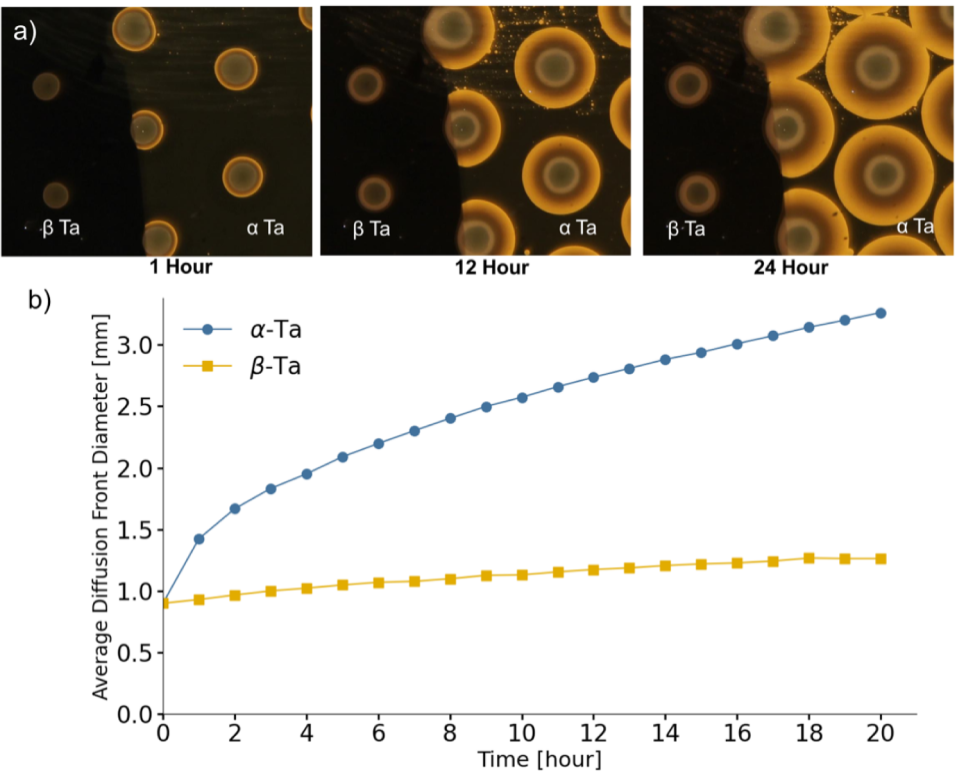
(a) Time-resolved optical images showing the hydrogen diffusion front expansion in the α-/β-Tantalum diffusion sample. Optical transmission images were captured at various time intervals (1, 12, and 24 h) after hydrogen exposure at 1 bar and 28 °C. Each brightening ring corresponds to the formation of yttrium hydride surrounding individual Pd catalyst dots, which locally dissociate H_2_ and initiate hydrogen uptake. b) Quantitative analysis of the average hydrogen diffusion front diameter as a function of time for both α-Ta and β-Ta, based on the optical images shown in Figure S16. Only data up to 20 h were analyzed, as the diffusion fronts of β-Ta overlapped beyond this point, making them difficult to distinguish.

The observation that hydrogen uptake in β-Ta is diffusion-limited, in contrast to the surface-limited kinetics observed in α-Ta, points to fundamental differences in hydrogen transport mechanisms between the two phases. As shown in Figure S17, α-Ta [space group Im3̅*m* (229)] adopts a simple body-centered cubic (bcc) structure with two atoms per unit cell. In this lattice, hydrogen atoms predominantly occupy tetrahedral interstitial (T) sites, which are energetically favorable, facilitating relatively fast diffusion.^25,42^ We know from our *in situ* neutron reflectometry measurements and previous in-plane *in situ* XRD studies that the lattice symmetry of β-Ta remains unchanged during hydrogen exposure.^25^ This suggests that hydrogen atoms occupy high-symmetry interstitial positions within the structure. β-Ta crystallizes in a complex tetragonal structure [space group *P*4̅2_1_ *m* (113)] with 30 Ta atoms per unit cell. The maximum multiplicity of the Wyckoff positions is 8,^43^ which is relatively low compared to the number of Ta atoms, implying that hydrogen atoms cannot be accommodated in a single unique site. Furthermore, neutron reflectometry measurements show that the hydrogen-to-tantalum ratio can reach up to β-TaH_0.5_,^25^ i.e., at least 16 sites within the unit cell will be occupied, supporting the idea that hydrogen atoms are likely distributed across multiple inequivalent interstitial sites. This structural complexity likely introduces multiple hydrogen diffusion pathways with varying energy barriers, which can impede long-range hydrogen mobility, especially at low temperatures.^39^ In addition to these lattice-related limitations, microstructural features such as grain boundaries may further affect hydrogen transport in β-Ta.^44^ As shown in Figure S18, XRD peak analysis reveals that the β-Ta studied here exhibits an average grain size of approximately 10 nm, compared to 14 nm for α-Ta, implying a higher density of grain boundaries in the β-phase. These boundaries may serve as trapping or scattering sites for hydrogen atoms, potentially hindering their transport. But, on the other hand, the increased grain boundary density may also enhance grain boundary diffusion.^39,44^

To optimize hydrogen transport in β-Ta and improve the response time, tuning the film microstructure presents a promising route. This can be achieved by adjusting deposition parameters such as substrate temperature, sputtering pressure, and power, all of which influence grain size, texture, and phase formation.^45,46^ For instance, higher substrate temperatures promote grain growth, while lower sputtering pressures lead to denser and more oriented films. Furthermore, the choice of substrate can affect phase stability and induce preferred grain orientations,^47^ both of which may critically impact hydrogen diffusion kinetics.

As a direction for further research, density functional theory (DFT) and molecular dynamics (MD) simulations could be instrumental in identifying the preferred hydrogen sites and mapping diffusion pathways in β-Ta, thereby providing insights into how microstructural engineering can be leveraged to accelerate hydrogen transport.

## 4 Conclusion

In this work, we investigated the structural and optical hydrogenation behavior of thin-film β-Ta under subzero conditions. *In situ* XRD measurements reveal a fully reversible lattice expansion without indications of phase transitions or plastic deformation, even at temperatures down to −60 °C. Compared to α-Ta, β-Ta exhibits a smaller hydrogen-induced lattice expansion, which is consistent with its lower hydrogen-to-metal ratio and reduced hydrogen solubility at room temperature.^25^

Optical transmission measurements demonstrate a hysteresis-free and monotoneous response over nearly 5 orders of magnitude in hydrogen pressure, with a temperature-independent resolution when normalized to a baseline pressure. This confirms that β-Ta maintains reliable sensing performance even in extreme environments. However, the response time of β-Ta increases significantly at low temperatures. Scaling analysis reveals that the hydrogenation kinetics are diffusion-limited unlike in α-Ta, where surface-limited dissociation governs the uptake. Direct visualization of hydrogen diffusion fronts in α- and β-Ta diffusion further corroborates this distinction.

These findings establish β-Ta as a structurally robust and optically stable hydrogen sensing material capable of operation down to −60 °C. While its response time is currently constrained by diffusion, this insight provides a clear direction for improvement. Future studies may focus on microstructural tuning, e.g., optimizing grain size and orientation through tailored deposition parameters, to enhance hydrogen diffusion and sensor performance. Overall, β-Ta emerges as a strong candidate for next-generation hydrogen sensors in demanding subzero environments such as aviation and cryogenic fuel systems.

## Supplementary Material



## Notes

Sputtering parameters of β-Ta and dual-phase α-/β-Ta thin films (Tables S1–S3); sample schematics; X-ray reflectivity data, fits, and scattering length density profiles; atomic force microscopy (AFM) morphology; scanning electron microscopy (SEM) micrographs; schematics of experimental setups for low-temperature XRD and optical measurements; additional XRD patterns before and after hydrogen exposure and cycling; normalized lattice expansion of β- and α-Ta; wavelength-dependent optical transmission spectra; optical hydrogen response and cyclability data; response time curves; optical diffusion front measurements in α-/β-Ta; crystal structure models; grain size analysis from XRD peak broadening; sensitivity analysis of optical response (PDF)
