# Supplementary material for: Structural Stability and Kinetics of Hydrogenation of β‑Tantalum at Low Temperatures
Source: J Phys Chem C Nanomater Interfaces. 2025 Oct 16;129(43):19292–302. doi: 10.1021/acs.jpcc.5c05265 (PMC12581347; doi:10.1021/acs.jpcc.5c05265)
Supplement: Supplementary file 1 [file jp5c05265_si_001.pdf]

Supporting Information
Structural Stability and Kinetics of Hydrogenation of β-Tantalum at Low-Temperatures

Ziqing Yuan,[†] Herman Schreuders,[†] Ewout Voorrips,[†] Robert Dankelman,[†] Roger M. Groves,[‡] Bernard Dam,[†] and Lars J. Bannenberg[*,†]

†*Faculty of Applied Sciences, Delft University of Technology, Mekelweg 15, 2629 JB Delft, The Netherlands*

‡*Faculty of Aerospace Engineering, Delft University of Technology, Kluyverweg 1, 2629 HS Delft, The Netherlands*

E-mail: l.j.bannenberg@tudelft.nl

Table S1: Sputtering settings for the β-Ta sample,as depicted in Fig. S1.

**Layer**	**Parameter**	**β-Ta**
Sensing Layer	Power Ta [W]	130
	Time [s]	140
Capping Layer	Power Pd [W]	51
	Power Au [W]	27
	Time [s]	26
PTFE Layer	Power [W]	70
	Time [min]	19

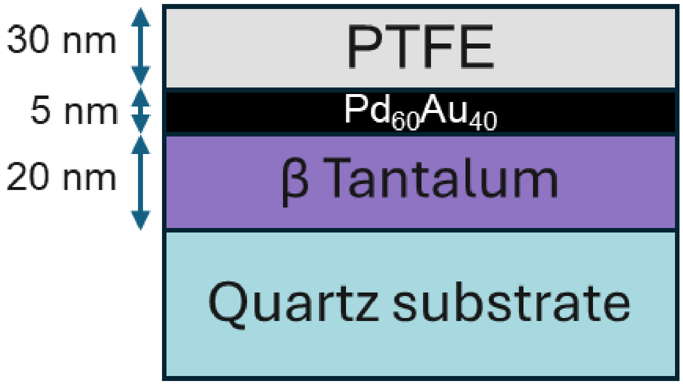

Figure S1: Sample schematic: The sample consists of a 20 nm β-Ta sensing layer, and a 5 nm $Pd_{60}Au_{40}$ capping layer, covered by a 30 nm PTFE layer.

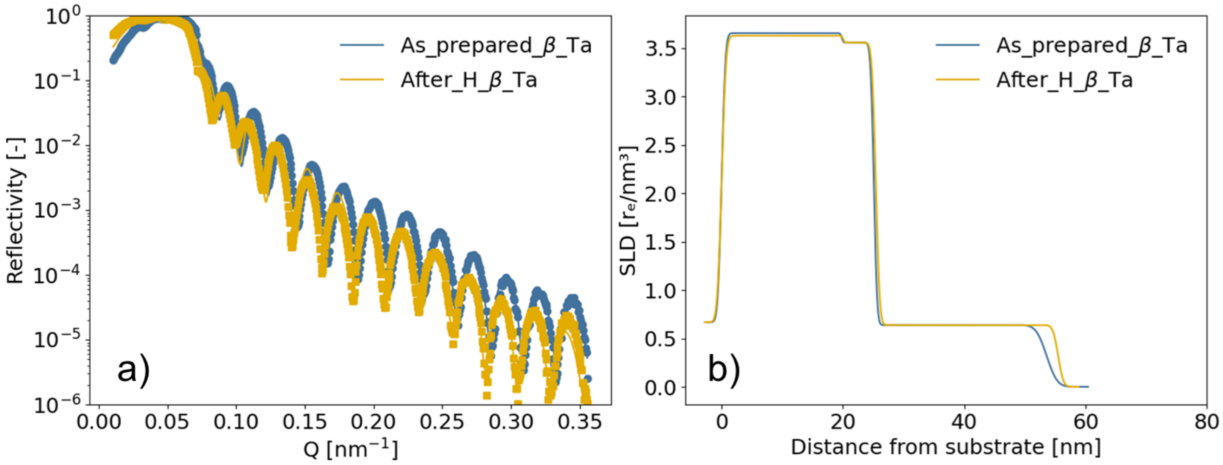

Figure S2: X-ray Reflectivity (XRR) data, a) corresponding fits, and b) Scattering Length Density (SLD) profiles for the same sample measured in the as-prepared state and after exposure to hydrogen, as indicated in the legend. The sample consists of a 20 nm β-Ta sensing layer, and a 5 nm $Pd_{60}Au_{40}$ capping layer, covered by a 30 nm PTFE overlayer (nominal thicknesses). The dots in panel a) represent the experimental XRR data, while the continuous lines are the fitted curves. Panel b) shows the depth-dependent SLD profiles derived from the fits.

Table S2: Fitted layer thickness, density, and roughness of the as-prepared and after-H samples with various compositions. The corresponding fits and SLD profiles can be found in Fig. S2. The density of the substrate was fixed to the literature value of 0.022 FU/Å^3, and the roughness was fitted for all samples. All thicknesses and roughness values are given in nanometers.

Parameters	As-prepared β-Ta	After-H β-Ta
PTFE Thickness [nm]	28.5	30.1
PTFE Roughness [nm]	1.4	0.7
PTFE Density [FU/Å^3]	0.0132	0.0132
PdAu Thickness [nm]	5.3	5.4
PdAu Roughness [nm]	0.4	0.5
PdAu Density [FU/Å^3]	0.062	0.062
Ta Thickness [nm]	19.8	20.1
Ta Roughness [nm]	0.2	0.2
Ta Density [FU/Å^3]	0.0544	0.0540
Substrate Density [FU/Å^3]	0.022	0.022
Substrate Roughness [nm]	0.5	0.6

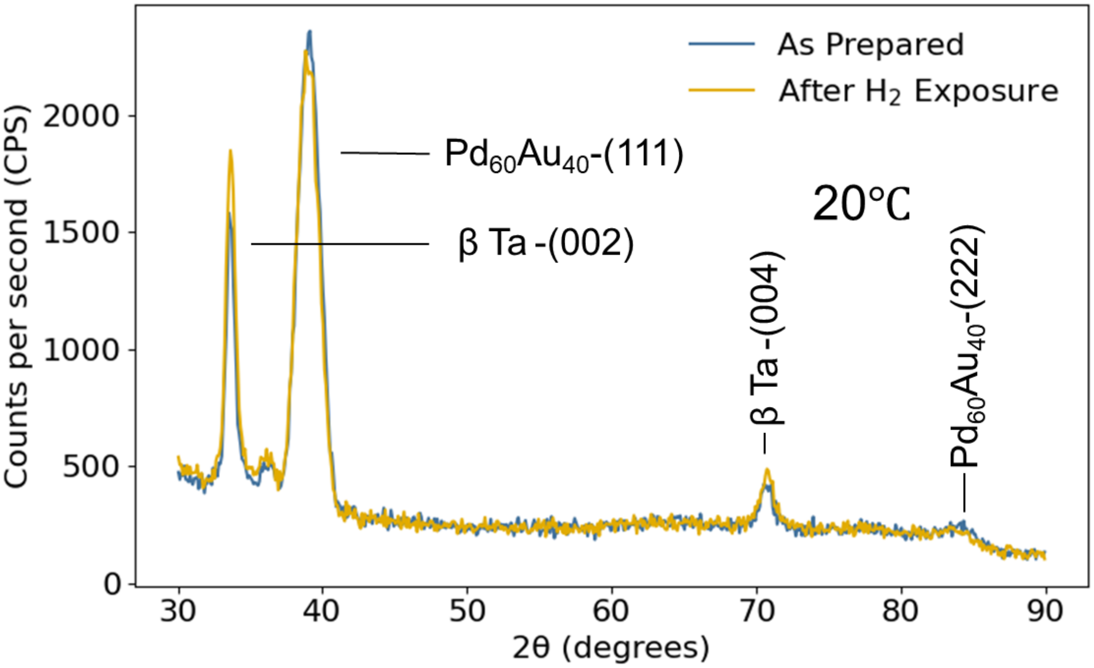

Figure S3: X-ray diffraction patterns of a 20 nm β-Ta thin film with a 5 nm $Pd_{60}Au_{40}$ capping layer and a 30 nm PTFE protective layer on a quartz substrate at 20°C for the same sample measured in the as-prepared state and after exposure to hydrogen, as indicated in the legend, showcasing the 30-90° range peaks. The diffraction pattern was obtained using Cu-Kα radiation ($\lambda = 0.1542$ nm).

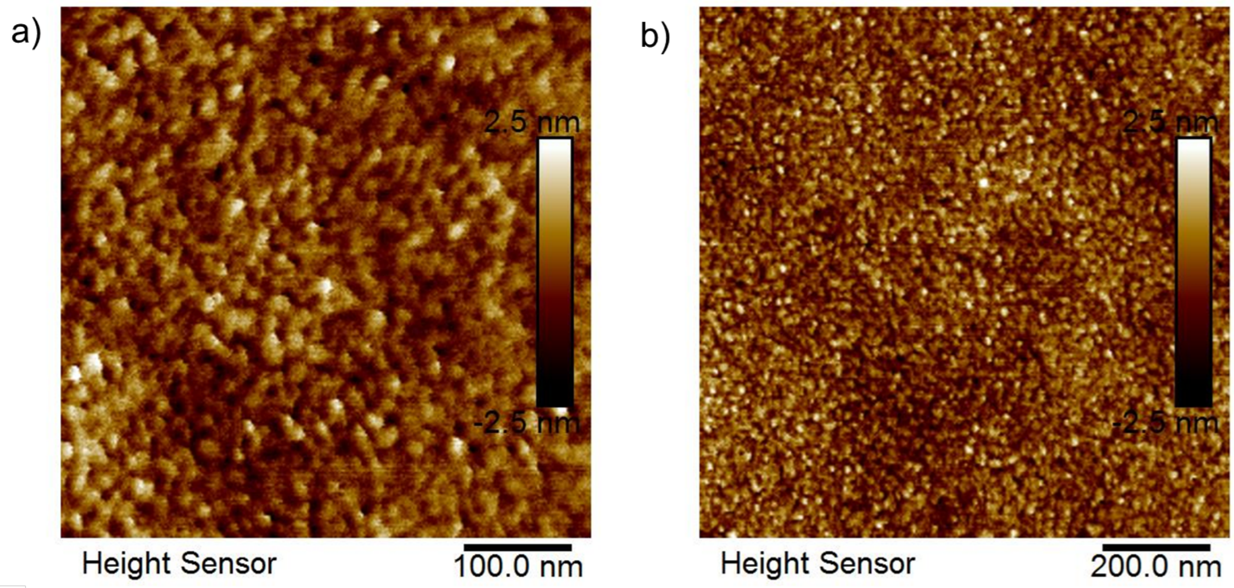

Figure S4: Tapping-mode AFM height images of the multilayer film, consisting of a 20 nm β-Ta sensing layer, a 5 nm $Pd_{60}Au_{40}$ catalyst layer, and a 30 nm PTFE protection layer, recorded over a) 100 $\times$ 100 nm^2 and b) 200 $\times$ 200 nm^2 scan areas.

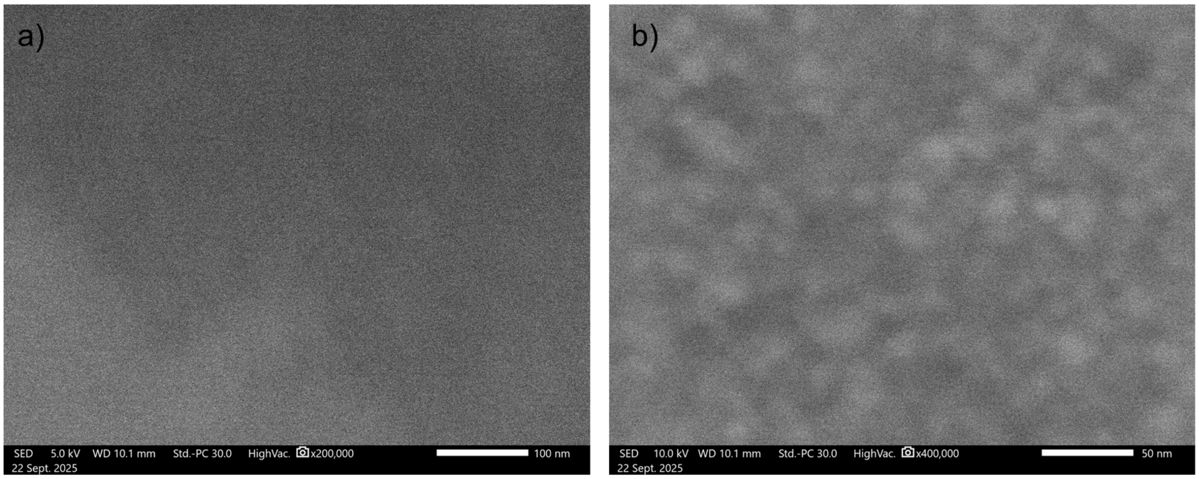

Figure S5: Scanning electron microscopy (SEM) micrographs of the multilayer thin films obtained using a JSM-IT 700HR (JEOL, Germany) in high-vacuum mode with a secondary electron detector (SED). a) 20 nm β-Ta sensing layer, a 5 nm $Pd_{60}Au_{40}$ catalyst layer, with a 30 nm PTFE capping layer. Images were acquired at accelerating voltages at 5 kV with magnifications up to 200,000 times. b) Reference sample without PTFE: 20 nm β-Ta sensing layer, a 5 nm $Pd_{60}Au_{40}$ catalyst layer. Images were acquired at accelerating voltages at 10 kV with magnifications up to 400,000 times.

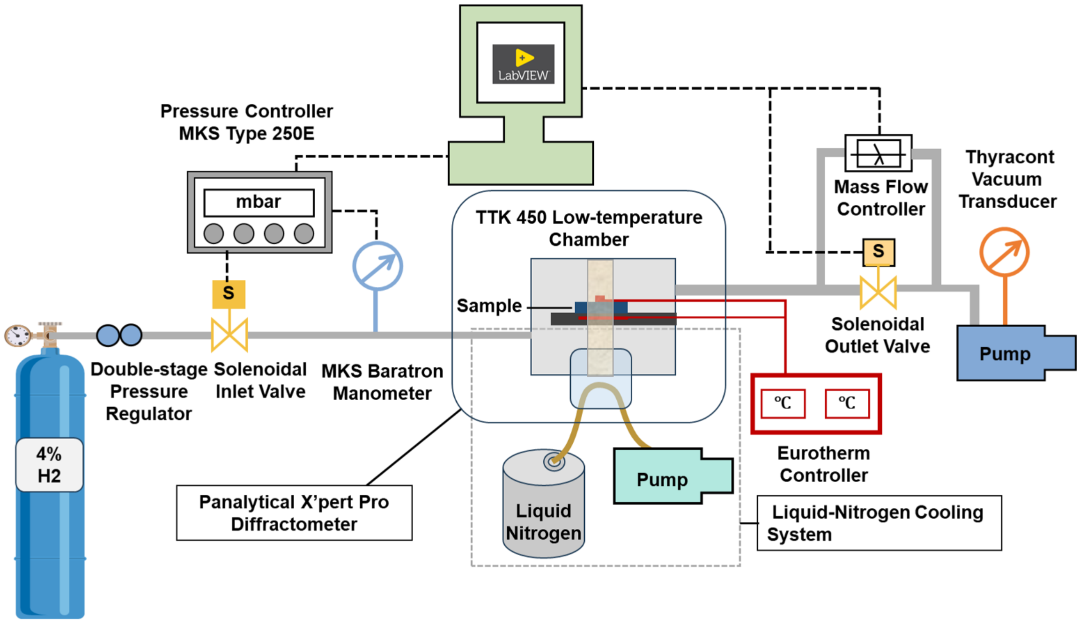

Figure S6: Schematic diagram of the pressure and temperature control system of the TTK 450 low-temperature chamber.

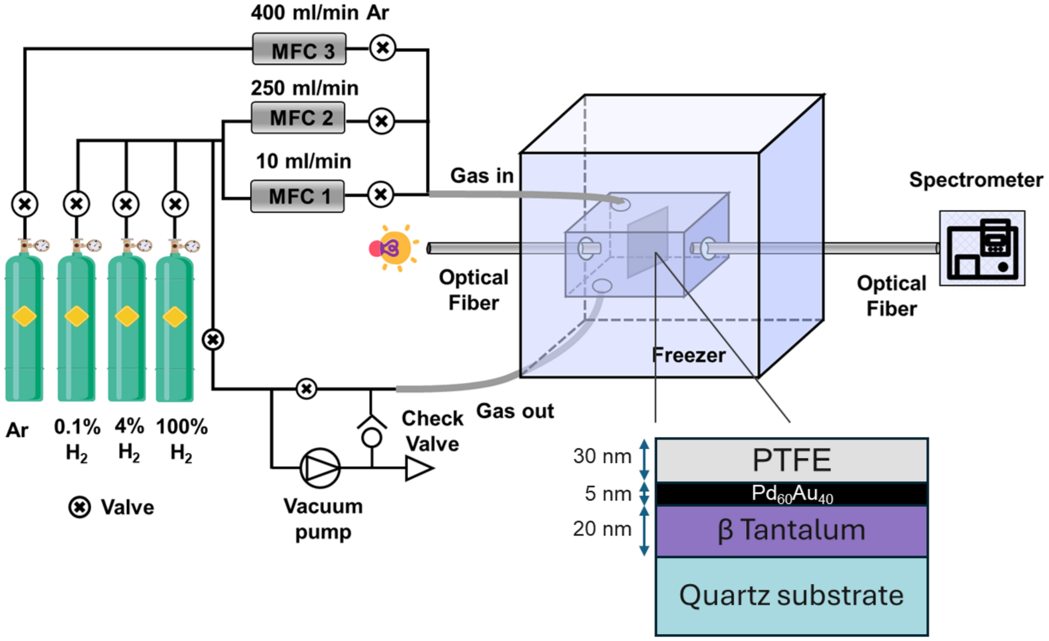

Figure S7: Schematic diagram of the experimental configuration designed for quantifying the change in transmittance of metal hydride thin films when subjected to varying hydrogen concentrations at low temperatures.

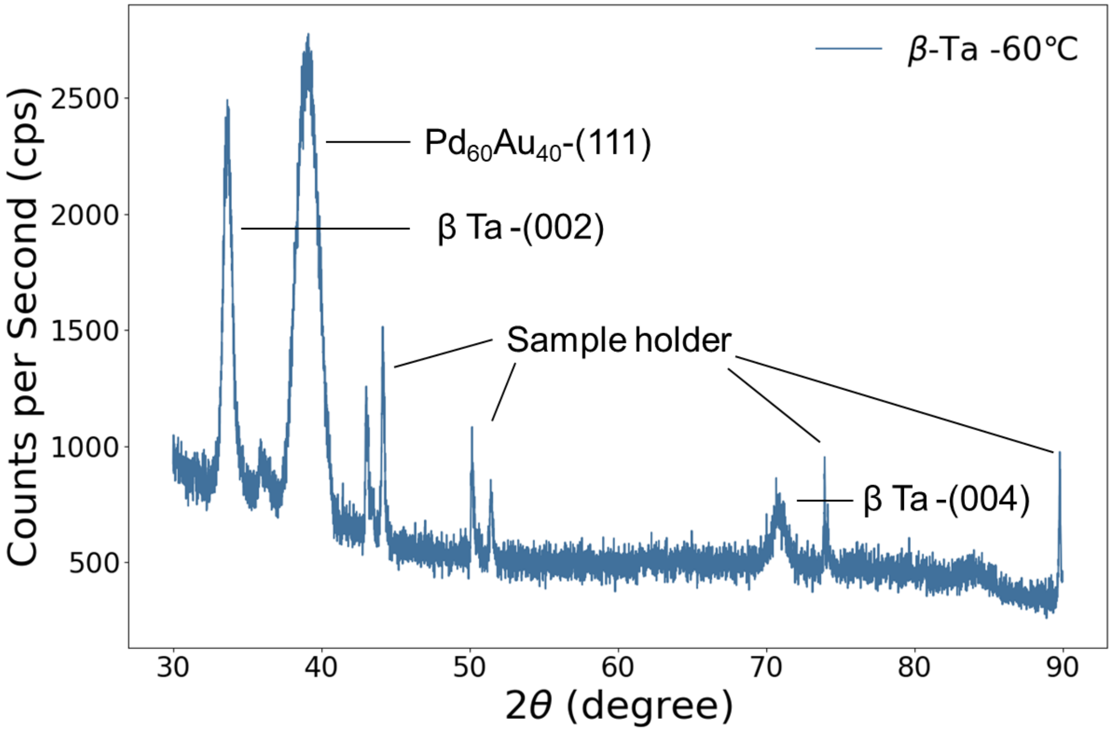

Figure S8: In situ X-ray diffraction (XRD) pattern of a 20 nm β-Ta thin film with a 5 nm $Pd_{60}Au_{40}$ capping layer and a 30 nm PTFE protective layer on a quartz substrate at -60°C without hydrogen, showcasing the 30-90° range peaks. Note that the background visible in the plot originates from the sample holder. The diffraction pattern was obtained using Cu-Kα radiation ($\lambda = 0.1542$ nm).

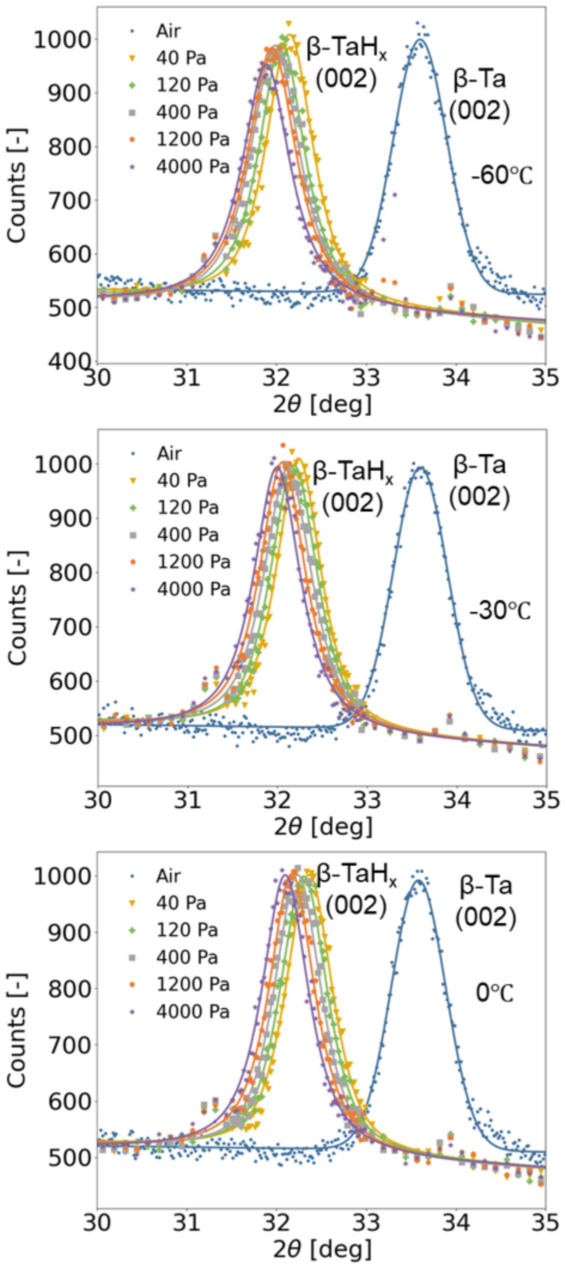

Figure S9: In situ X-ray diffraction patterns of a 20 nm β-Ta thin film with a 5 nm $Pd_{60}Au_{40}$ capping layer and a 30 nm PTFE protective layer on a quartz substrate for the partial hydrogen pressures indicated in the legend and measured at -60°C, -30°C, and 0°C. The continuous lines represent fits of the pseudo-Voigt functions to the experimental data.

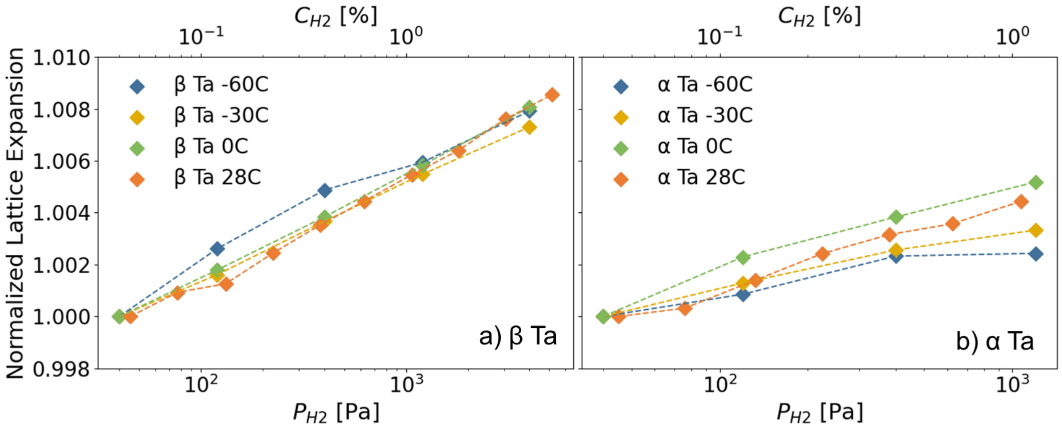

Figure S10: Normalized lattice expansion of a) β-Ta and b) α-Ta thin films as a function of partial hydrogen pressure (P_{H_2}) at various temperatures (-60°C, -30°C, 0°C, and 28°C). The expansion is referenced to the lowest measured P_{H_2}. The dashed lines are guides to the eye.

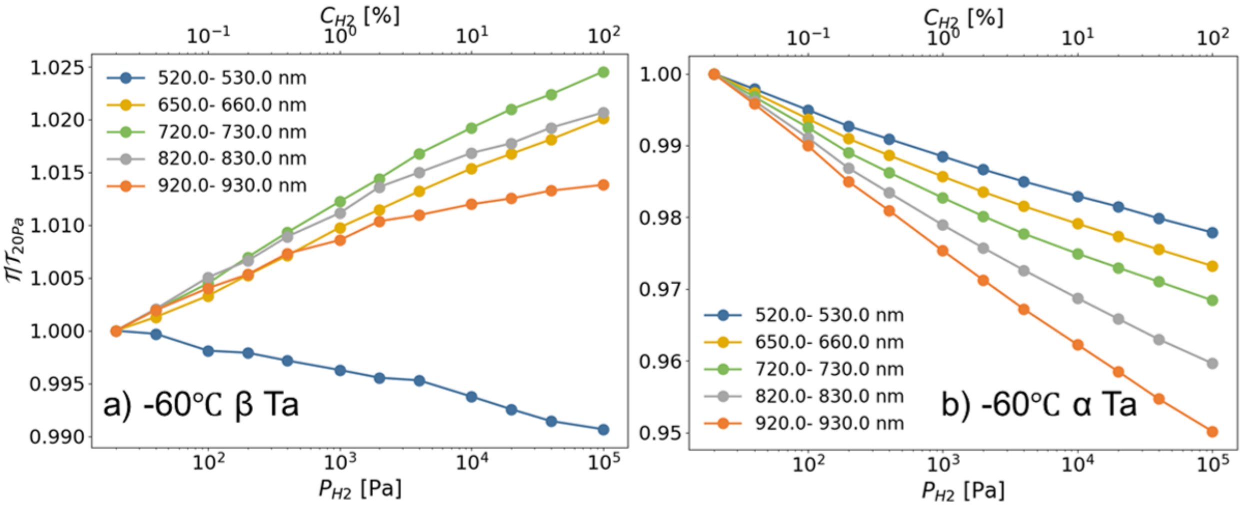

Figure S11: Wavelength-dependent optical transmission response of a) β-Ta and b) α-Ta thin films at -60°C as a function of partial hydrogen pressure (P_{H_2}). The normalized transmission ($\mathcal{T}/\mathcal{T}_{20Pa}$) is referenced to the lowest measured pressure ($P_{H_2} = 20$ Pa).

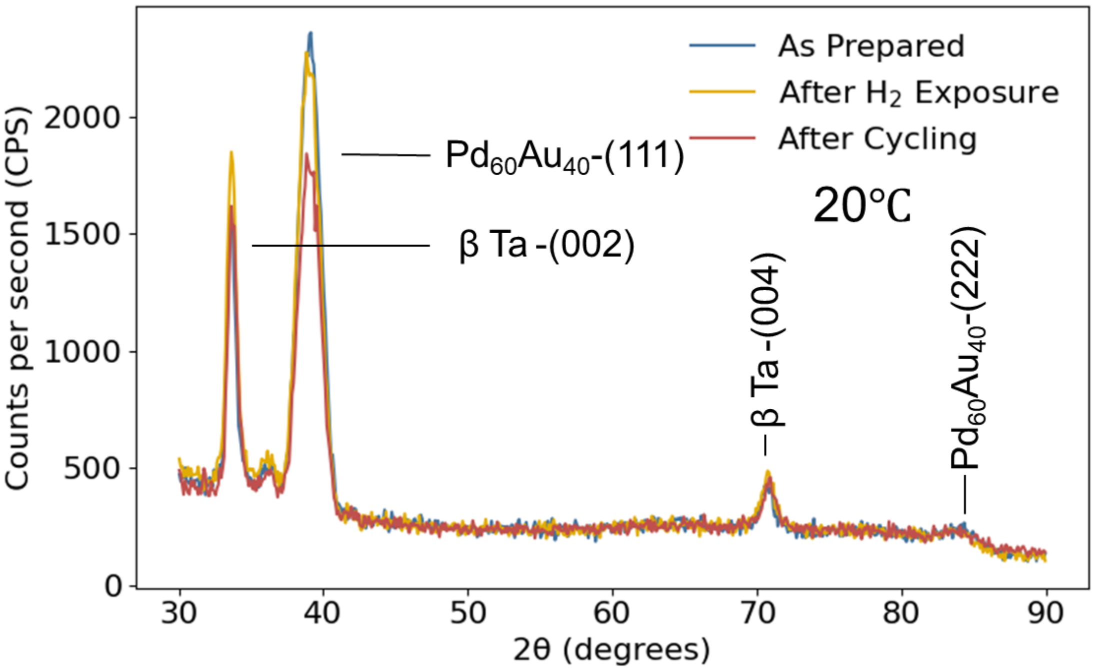

Figure S12: X-ray diffraction patterns measured at 20°C for the same sample in the as-prepared state, after exposure to hydrogen, and after cycling, as indicated in the legend, showcasing the 30-90° range peaks. The sample consists of a 20 nm β-Ta thin film with a 5 nm $Pd_{60}Au_{40}$ capping layer and a 30 nm PTFE protective layer on a quartz substrate. The diffraction patterns were obtained using Cu-Kα radiation ($\lambda = 0.1542$ nm).

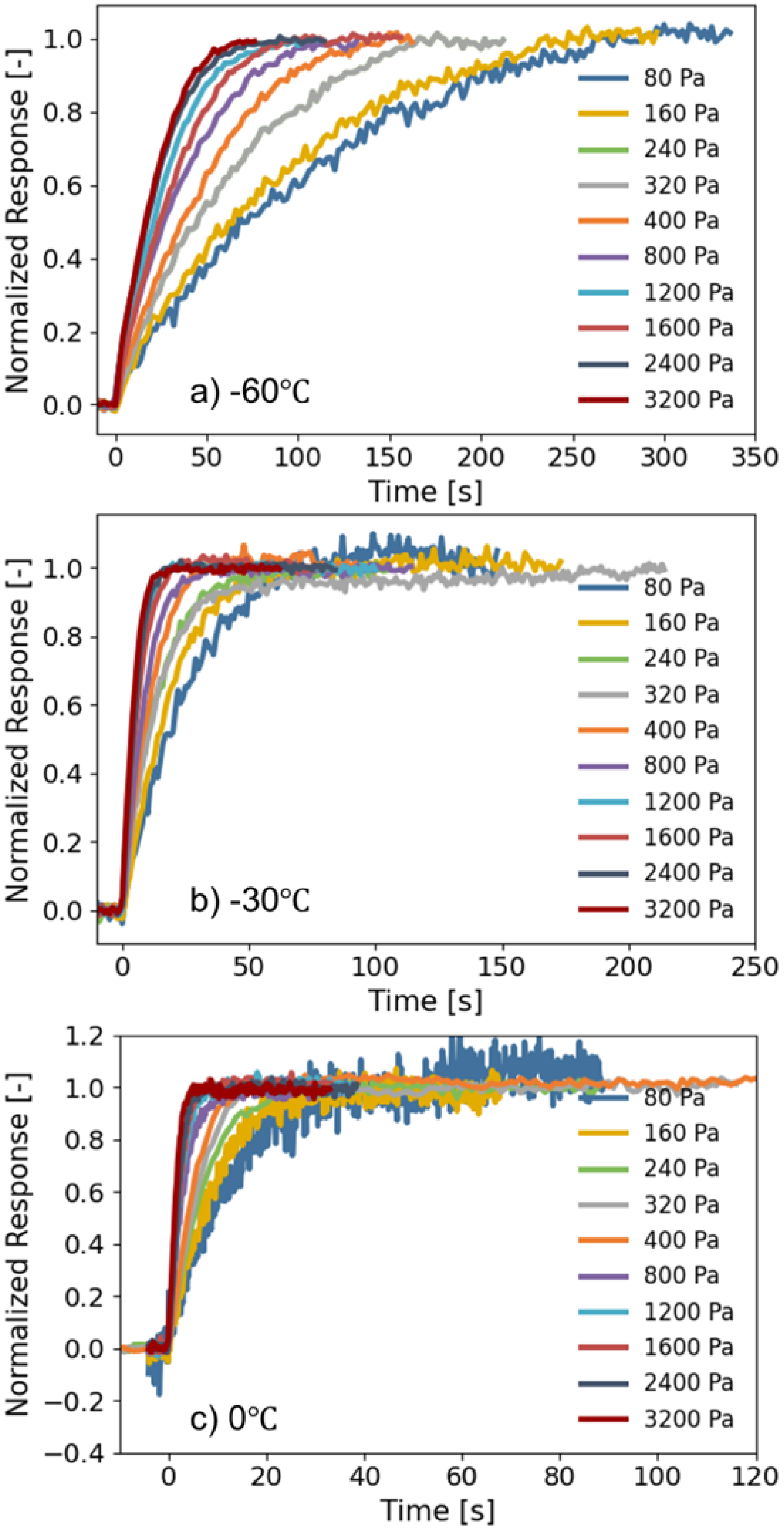

Figure S13: Normalized responses of β-Ta at a) -60°C, b) -30°C and c) 0°C to various hydrogen pressures ranging from 80 Pa to 3200 Pa.

Table S3: Sputtering settings for dual-phase (α-Ta / β-Ta) sample.

Layer	Parameter	Value
Ti Seed Layer (only α-Ta side)	Power Ti [W]	100
	Time [s]	126
Ta Layer (full wafer)	Power Ta [W]	130
	Time [s]	570
Y Capping Layer (full wafer)	Power Y [W]	75
	Time [s]	409
Pd Dots	Power Pd [W]	50
	Time [s]	180

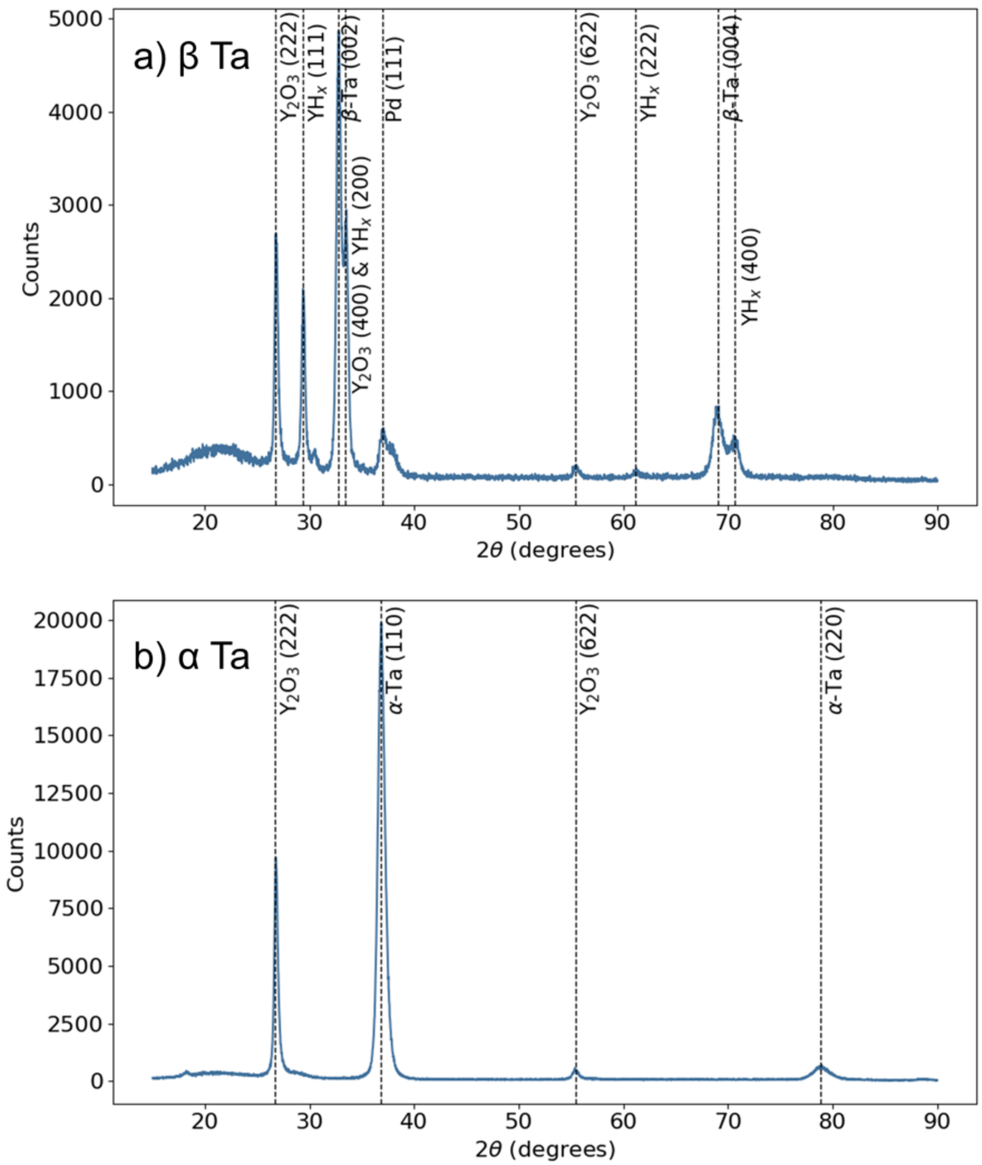

Figure S14: X-ray diffraction (XRD) patterns of the dual-phase Ta sample. a) β-Ta region deposited directly on the quartz substrate, showing characteristic reflections including (002) and (004), as well as weaker peaks attributed to Pd catalyst and surface-oxidized Y_2O_3. b) α-Ta region on the Ti-coated half, displaying distinct bcc reflections of (110), and (220), along with contributions from the oxidized Y indicator layer. These data confirm the successful formation of separate α-Ta and β-Ta phases as designed. **Note:** the measurement was conducted within 24 hours after exposure to hydrogen.

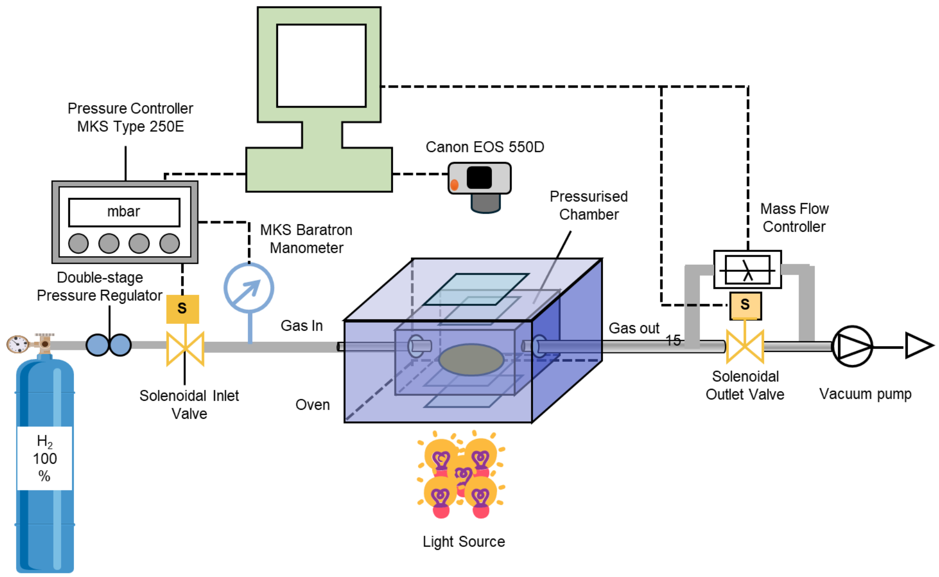

Figure S15: Schematic of the optical setup used to measure in-plane hydrogen diffusion in the α-/β-Tantalum diffusion sample. Hydrogen diffusion experiments were performed at 28°C and a hydrogen partial pressure of 1 bar ($C_{\mathrm{H}_2} = 100\%$), using an optical transmission setup similar to the hydrogenography system. The setup consists of a pressure-controlled chamber equipped with top and bottom optical windows, enabling illumination and imaging of the sample. The sample is placed horizontally on a flat holder connected to gas inlets, a venting line, and a pressure control unit (MKS Type 250E, Andover, MA, USA), with pressure monitored via a Baratron manometer (MKS 120AD, Andover, MA, USA). Illumination is provided from below by several visible-light lamps positioned approximately 40 cm beneath the sample, while a Canon EOS 550D camera equipped with an 18–135 mm lens is mounted above to capture images at fixed time intervals. After recording a reference image under vacuum, hydrogen gas is introduced to a total pressure of 1 bar and maintained throughout the experiment. Hydrogen uptake is visualized as a circular brightening front surrounding each Pd dot, corresponding to the transformation of Y into an optically absorbing hydride.

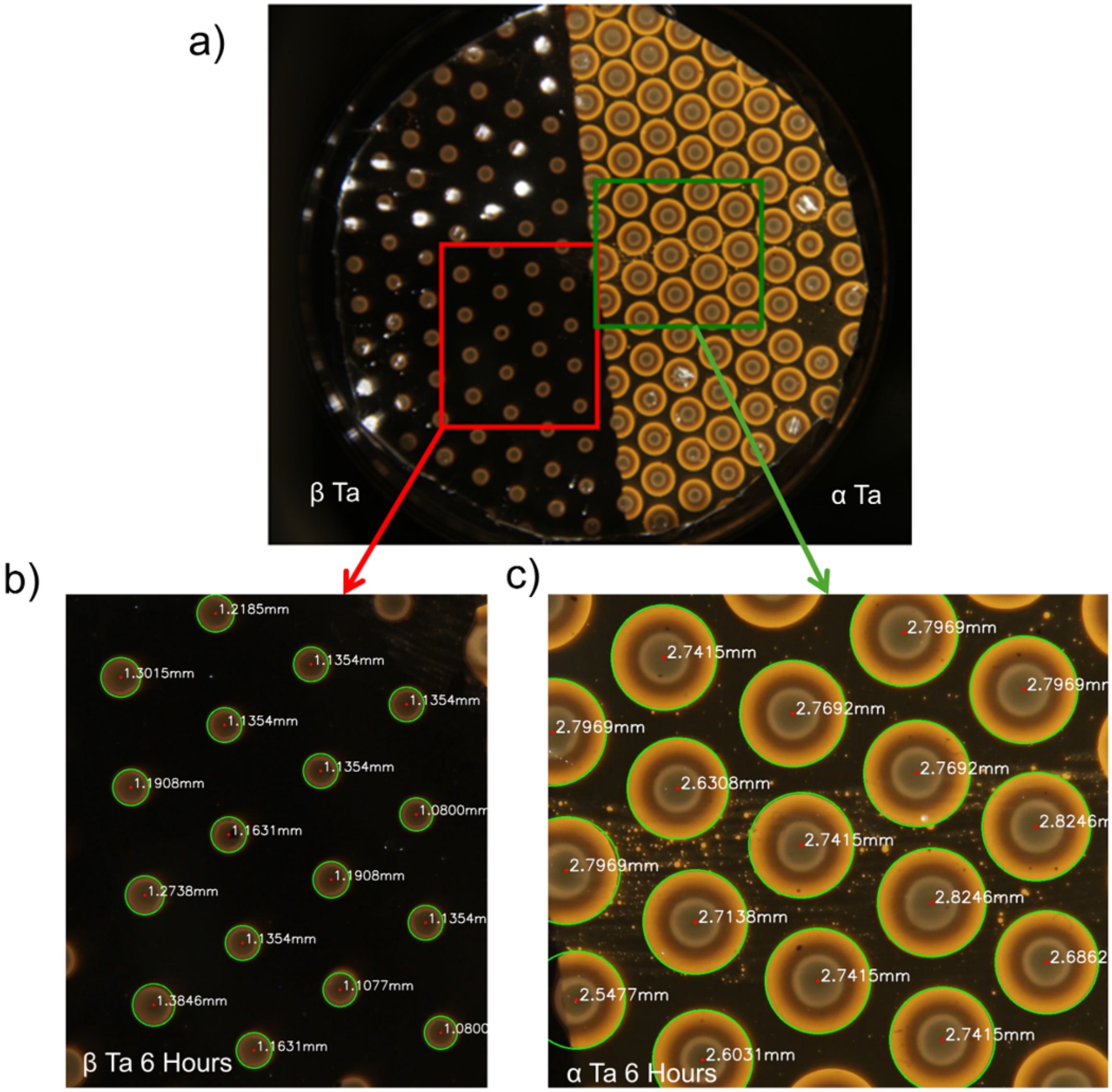

Figure S16: a) Representative optical image of the Ta diffusion sample showing both β-Ta (left) and α-Ta (right) regions. The red and green boxes highlight the areas selected for further analysis. This image was acquired from a central region of the sample to minimize lens distortion. b,c) Cropped regions from the β-Ta and α-Ta sides, respectively, after 6 h of hydrogen exposure. The hydrogen diffusion front diameters were quantitatively extracted using a custom Python script based on OpenCV. Pixel-to-length calibration was performed using a scale reference in the original image. These measurements support the diffusion front evolution analysis shown in Fig. 8b of the main text.

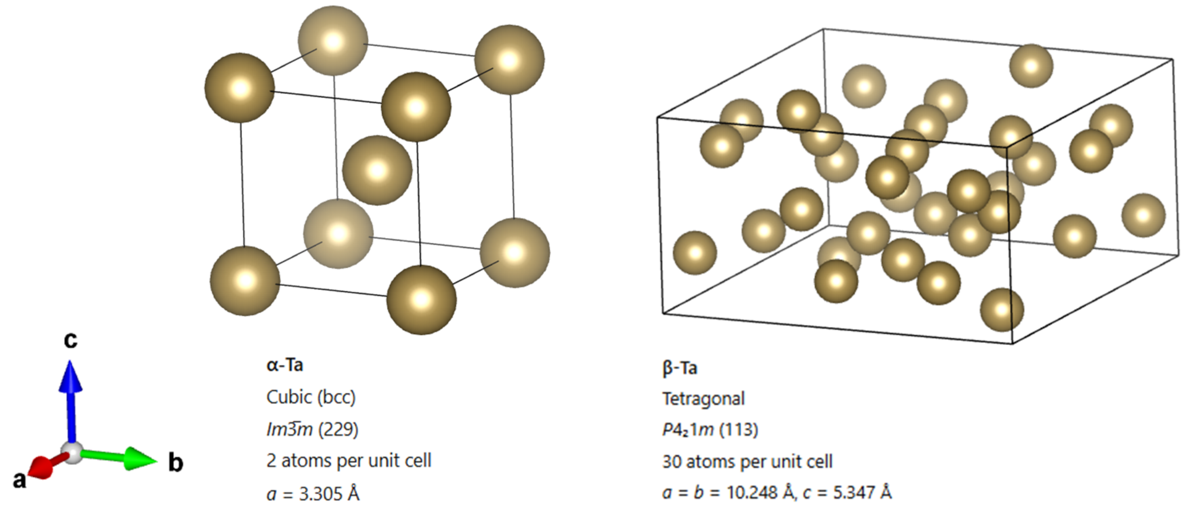

Figure S17: Crystal structures of a) α-Ta and b) β-Ta.[1,2]

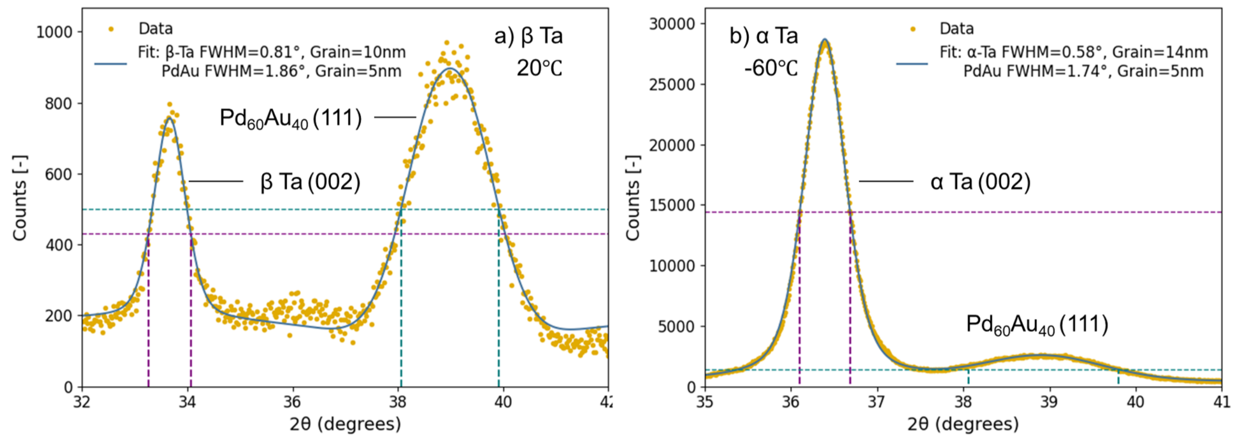

Figure S18: Full-width at half-maximum (FWHM) values extracted from X-ray diffraction peak fitting of α-Ta and β-Ta thin films. The FWHM values were obtained by pseudo-Voigt profile fitting of the main diffraction peaks. The average grain size was estimated using the Scherrer equation, $D = \frac{K\lambda}{\beta \cos\theta}$, where $K = 0.9$ is the shape factor, $\lambda = 0.1542\,\mathrm{nm}$ is the X-ray wavelength (Cu Kα), β is the FWHM in radians, and θ is the Bragg angle.

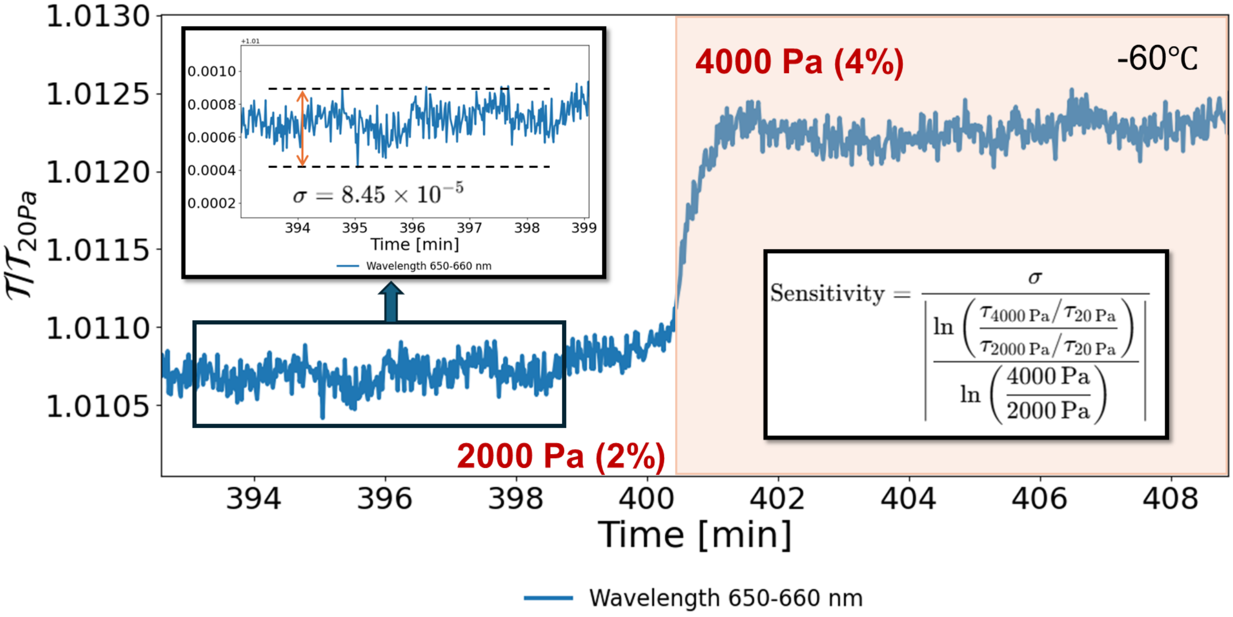

Figure S19: Sensitivity analysis of the optical transmission signal ($\mathcal{T}/\mathcal{T}_{20\,\mathrm{Pa}}$) measured at 650–660 nm under hydrogen exposure at -60°C. The standard deviation $\sigma = 8.45 \times 10^{-5}$ is calculated from the steady-state 2% H_2 region (393–399 min), and the optical response between 2% (2000 Pa) and 4% (4000 Pa) H_2 is used to determine the sensor's sensitivity. The corresponding formula for sensitivity is shown in the inset, yielding a value of $\Delta P_{\mathrm{H}_2}/P_{\mathrm{H}_2} \approx 0.038$ that is relatively constant with pressure, that is, substantially better than the most stringent requirement of 5% set by the U.S. Department of Energy. The data were acquired with a scan-to-average setting of 500, using an integration time of 4 ms per spectrum (equivalent to an effective sampling frequency of 0.5 Hz).

References

(1) Jain, A.; Ong, S. P.; Hautier, G.; Chen, W.; Richards, W. D.; Dacek, S.; Cholia, S.; Gunter, D.; Skinner, D.; Ceder, G.; Persson, K. A. Commentary: The Materials Project: A materials genome approach to accelerating materials innovation. *APL Materials* **2013**, *1*, 011002.

(2) Momma, K.; Izumi, F. VESTA 3 for three-dimensional visualization of crystal, volumetric and morphology data. *Journal of Applied Crystallography* **2011**, *44*, 1272–1276.
